# Padres Preparados, Jóvenes Saludables: intervention impact of a randomized controlled trial on Latino father and adolescent energy balance-related behaviors

**DOI:** 10.1186/s12889-022-14284-5

**Published:** 2022-10-18

**Authors:** A. Baltaci, G. A. Hurtado Choque, C. Davey, A. Reyes Peralta, S. Alvarez de Davila, Y. Zhang, A. Gold, N. Larson, M. Reicks

**Affiliations:** 1 Division of Epidemiology and Community Health, School of Public Health, University of Minnesota, 1300 S Second St, Suite 300, Minneapolis, MN 55454 USA; 2 School of Public Health, University of Maryland, 4200 Valley Dr, College Park, MD 20742 USA; 3 Clinical and Translational Science Institute, University of Minnesota, 717 Delaware Street SE, Minneapolis, MN 55414 USA; 4 Center for Family Development, University of Minnesota Extension, 1420 Eckles Ave, St. Paul, MN 55108 USA; 5 Center for Family Development, University of Minnesota Extension, Robert J. Jones Urban Research and Outreach-Engagement Center, 2001 Plymouth Ave N, Minneapolis, MN 55411 USA; 6 Department of Child and Adolescent Health and Social Medicine, School of Public Health, Medical College of Soochow University, 199 Ren Ai Road, Building 401, Room 1411, Suzhou, 215123 Jiangsu China; 7 Department of Food Science and Nutrition, University of Minnesota, 1334 Eckles Ave, St. Paul, MN 55108 USA

**Keywords:** Child obesity prevention, Community-based intervention, Randomized control trial, Intervention, Latino adolescents, Latino fathers, Energy balance-related behaviors

## Abstract

**Background:**

Studies have shown associations among food and activity behaviors and body weight of Latino fathers and adolescents. However, few Latino father-focused interventions have been designed to improve energy balance-related behaviors (EBRBs) and weight status among early adolescents. Thus, this efficacy study aims to evaluate the Padres Preparados, Jóvenes Saludables (Padres) youth obesity prevention program for positive changes in EBRBs (fruit, vegetable, sugar-sweetened beverage (SSB), sweet/salty snack, and fast-food consumption, physical activity, and screen time) and weight status among low-income Latino fathers and adolescents (10-14 years).

**Methods:**

A two-arm (treatment versus delayed-treatment control group) randomized controlled trial was conducted to evaluate the efficacy of 8 weekly experiential learning sessions (2.5 hours each) based on social cognitive theory. The sessions included food preparation, parenting skills, nutrition, and physical activity. The program was delivered to father-adolescent dyads (mothers were encouraged to attend) in trusted community-based settings in a Midwest metropolitan area between 2017 and 2019. In March 2020, in-person implementation was discontinued due to COVID-19 pandemic restrictions, which limited the sample size. Father/adolescent dyads were randomized to treatment or control group within each site. Surveys and measurements were completed by fathers and adolescents to assess changes in food and activity behaviors from baseline to post-intervention. Adolescents also completed 24-hour dietary recall interviews at baseline and post-intervention. Intervention effects were assessed using linear regression mixed models adjusted for covariates and accounting for clustering of participants within sites.

**Results:**

Data from 147 father/adolescent dyads who completed at least the baseline data collection were used. No significant differences were observed for baseline to post-intervention changes in adolescents’ and fathers’ EBRBs or weight status between treatment and control groups. Fathers’ SSB and fast food intakes were not statistically significant (*p* = 0.067 and *p* = 0.090, respectively).

**Conclusions:**

The Padres program resulted in no significant improvements in adolescent and father EBRBs and weight status. Additional Latino father-focused interventions are needed to examine intervention effects on EBRBs among Latino adolescents.

**Trial registration:**

The Padres Preparados, Jóvenes Saludables study is registered with the U.S. National Library of Medicine, ClinicalTrials.gov Identifier: NCT03469752 (19/03/2018).

**Supplementary Information:**

The online version contains supplementary material available at 10.1186/s12889-022-14284-5.

## Background

Childhood obesity is a major public health concern. U.S. nationally representative data (2017-2018) indicate that the prevalence of obesity among children and adolescents (2-19 years) by Mexican American and Hispanic origin is 25.6 and 26.9%, respectively [1]. Childhood obesity is a health risk factor that can affect adult obesity, diabetes, metabolic disorders, and heart disease [2, 3]. In addition, children with overweight/obesity can experience lower self-esteem, a higher likelihood of being bullied, lower school attendance levels and performance, and fewer job prospects and lower-paid employment as an adult compared to children with a BMI below the 85th percentile [3]. Individual, socioeconomic, and environmetal factors that increase the risk of childhood obesity include calorie dense, nutrient poor food choices, sedentariness, low caregiver education, household poverty, lack of access to physical activity resources, and living in a food desert [4].

The behavioral determinants of excessive weight gain and obesity for children and adolescents include energy balance-related behaviors (EBRBs), namely high intake of energy-dense foods, low levels of physical activity, and frequent screen time [5, 6]. Mexican American and other Hispanic children, like many Americans, under consume fruits and vegetables [7, 8] and have excessive intakes of sugar-sweetened beverages (SSBs) [9], sweet/salty snacks [10], and fast food [11] compared to Dietary Guidelines for Americans (DGAs) recommendations [12, 13]. Mexican American and other Hispanic children also have lower levels of physical activity and higher levels of screen time than U.S. national recommendations [14–17]. National data also show that US Hispanic/Latino adults have unhealthy dietary behaviors [18] that do not meet DGA recommendations. Only about half (51.1%) of U.S. Hispanic/Latino men and one third of Hispanic/Latino women (31.3%) have adequate levels of moderate to vigorous physical activity per week [19] based on U.S. national recommendations [15].

Family resilience, which refers to the ability to meet challenges to effective functioning, has four domains related to the health and wellbeing of Latino families, including individual factors, family strengths, cultural values, and community support [20]. Familism is a well-studied cultural value that has been identified as a protective factor for Latino families because of its association with family cohesion, functioning, and communication [20, 21]. Parents (including fathers and other male caregivers) can play a crucial role in preventing obesity through the development of healthy food- and activity-related behaviors among adolescents [22]. Thus, focusing on the protective factors such as familism and family resilience in obesity prevention interventions with Latino families can foster positive parental involvement in EBRBs by both mothers and fathers.

Yet, most studies primarily focus on mothers when examining parental influence on adolescent’s diet with little attention on the role of the father [23]. Qualitative studies with Latino mothers [24, 25], fathers [26], and both mothers and fathers [27, 28] indicate that participants perceive mothers as having the primary responsibility for their children’s dietary behaviors, which may influence the level of fathers’ involvement. A review of qualitative and cross-sectional studies indicated that Latino fathers are more engaged in children’s physical activity related behaviors than dietary behaviors [29].

Latino fathers are underrepresented in existing obesity prevention interventions that promote a healthy lifestyle for Latino families [30]. Several studies indicate child and adolescent EBRBs are influenced by father food- and activity-related parenting practices [31, 32]. Positive associations are observed between fathers’ and children’s body weight, food intake, physical activity, and screen time [33–35]. Many interventions have been conducted to prevent childhood obesity with varying levels of parent involvement [36–38]. Some have targeted the food- and activity-related parenting practices of Latino parents [39–41]. Fewer interventions overall have focused primarily on fathers and their influence on the obesogenic behaviors of children and adolescents [42].

The Padres Preparados, Jóvenes Saludables (Padres) program was developed and implemented from 2017 to 2019 to address the lack of childhood obesity prevention interventions focused on Latino fathers in low-income families. The Padres program was adapted from an existing successful community-based parenting skills education program to prevent substance use among Latino parents and adolescents [43]. Community partners and Latino parents [43] had requested additional education programs to address obesity prevention among Latino early adolescents. The format, length, session structure, and content of the Padres program were designed based on Latino fathers’ feedback related to their beliefs, parenting experiences, and program preferences provided in father advisory board meetings and focus group discussions [44]. Consistent with the principles of community-based participatory research (CBPR) [45], community partners were engaged with researchers in all steps of the program design and implementation process. The Padres program curriculum was based on social cognitive theory [46, 47] because health and lifestyle behaviors are influenced by personal, social, and environmental factors [46, 47]. For low-income families, these factors may include availability of community resources, family culture, education, social norms, employment, and chronic life stressors [48]. The purpose of the current study is to assess whether Latino father and adolescent EBRBs, father BMI, and adolescent BMI percentile differed from baseline to post-intervention after the Padres program. The primary hypothesis was that father and adolescent EBRBs will be improved in the treatment group compared to the delayed-treatment control group based on baseline and post-intervention assessment.

## Methods

### Study design and sample

Padres Preparados, Jóvenes Saludables - Prepared Parents, Healthy Youth, was a community-based intervention project implemented in-person at four locations in the Minneapolis/St. Paul metropolitan area between September 2017 and December 2019 [49]. The randomized controlled intervention trial aimed to improve individual, social, physical, and environmental factors related to fathers’ and adolescents’ EBRBs to prevent overweight and obesity among Latino adolescents (10-14 years) (See Fig. S1 Padres Preparados Jovenes Saludables Program Trial Protocol for a more detailed description about the study intervention).

Flyers, announcements, and social media were used to recruit participants at community service centers and churches. Participants were Latino fathers or male caregivers with an adolescent (10-14 years). Eligibility criteria for fathers/caregivers were identifying as Latino, speaking Spanish, and having meals at least three times a week with their adolescent. While fathers and adolescents were the primary research participants, mothers were encouraged to attend sessions and complete data collection procedures. Fathers and mothers provided consent and adolescents provided assent to participate in the study. Fathers and adolescents received separate cash compensation for their participation. The study protocol was approved by the University of Minnesota Institutional Review Board and retrospectively registered at ClinicalTrials.gov: Identifier NCT03469752).

Self-administered surveys were completed in-person by fathers in Spanish and by youth in English at baseline and post-data collection (one week after eight sessions) with height and weight measured for all participants. In addition, 24-hour dietary recall interviews were conducted with youth at baseline and post-data collection to determine intervention effectiveness.

Following baseline data collection, father/adolescent dyads were randomized to an intervention or delayed-treatment control group. SAS randomization procedures were used to randomize participant dyads to intervention or delayed-treatment control within each site; a separate permuted block randomization schedule with block sizes of 2, 4, and 6 was generated for each site to ensure balance across treatment groups.

Random assignments were printed on colored paper slips and placed in sequentially numbered opaque envelopes to be distributed to enrolled families by the project coordinator. Randomization assignments were not consistent with group participation for eleven dyads due to misinterpretation of the group assignment or preferences of participants. In this analysis, father and adolescent data were analyzed according to their randomization group, regardless of the group in which they actually participated.

Father and youth dyads randomized to the intervention condition participated in the program immediately. Father and youth dyads randomized to the delayed-treatment control condition participated in the program 3 months after the post-data collection. Researchers and participants were not blinded to intervention and delayed-treatment control condition assignment.

### Study intervention

**Fig. 1:**
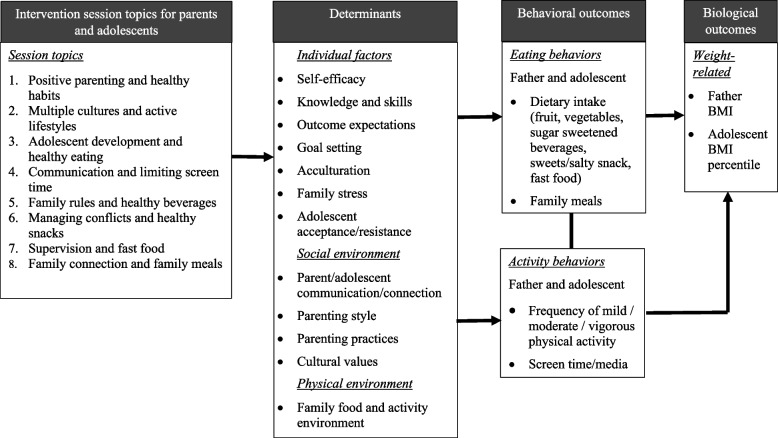
Padres Preparados, Jóvenes Saludables Program Conceptual Model

The Padres program curriculum consisted of eight in-person, 2.5-hour weekly group sessions based on a conceptual model described in Fig. 1. Parents and adolescents participated in skill-building activities together and separately in parent only, youth only, or parent/youth joint activities (See Table S1 for a more detailed description). Each session included food preparation, eating a meal together, parenting skills education, nutrition/physical activity education (together and separately), and physical activity (together). Participants prepared culturally tailored, simple recipes to support the nutrition concepts included in the sessions. Group physical activities were those that could be done easily indoors or outdoors regardless of time and resource constraints. Education for parents focused on parenting skills related to parent-child interactions and food- and activity-related parenting practices. Education for youth focused on EBRBs and building strong family communication and connections. Parent and youth joint activities involved explanations of basic nutrition and physical activity concepts and hands-on practice/discussion based on their experiences. The intervention highlighted healthy eating and physical activity and their associations with overall health instead of weight loss (Table S2 for a more detailed description). Discussion guide handouts and take-home activity sheets were provided at each session.

Parent intervention group sessions were delivered in Spanish by two trained bilingual facilitators who were parents themselves (one male and one female). Facilitators were Extension educators, community partner staff members, or participants in previous Extension parenting classes. Facilitators for the parent group sessions were trained by the project coordinator and two bilingual Extension and community educators who were involved in curriculum development and regularly met with the project team. Youth intervention group sessions were taught in English by two trained community partner staff members or graduate or undergraduate research assistants. The project coordinator and project team graduate research assistants trained youth facilitators.

### Sociodemographic characteristics

Fathers completed surveys to provide information about their age, years in the U.S., education, employment status, marital status, annual family income, and language spoken at home. Adolescents reported their birthdates and sex.

Adolescent’s age at baseline was calculated as the difference between birthdate and baseline data collection date divided by number of days in each year (365; 366 for leap years). Father education was classified as middle school or lower, general education development test that shows high school academic knowledge (GED) or high school, and some college or higher. Employment was collapsed into four categories: self-employed, unemployed, part-time employed, and full-time employed. Marital status was classified as single, married, or with a partner.

Food security was assessed by asking fathers the following questions from the USDA Food Security Module [50] “Within the past 12 months, we worried about whether our food would run out before we got money to buy more” and “Within the past 12 months, the food we bought just didn’t last and we didn’t have money to get more.” Response options were often true, sometimes true, never true. If fathers responded often or sometimes true to one of the two questions, they were classified as food insecure.

The question that assessed language spoken at home had five response options as “Spanish only, more Spanish than English, almost equal amount of Spanish and English, more English than Spanish, and English only.” Language spoken at home was categorized and coded as exclusively or primarily Spanish = 0, equally Spanish and English = 1, and more English than Spanish or only English = 2. The years in the U.S. variable was classified and coded into four categories: < 10 years = 0, ≥ 10 - < 20 years = 1, ≥ 20 - < 30 years = 2, and ≥ 30 years = 3.

### Measurements

Behavioral outcomes for fathers and adolescents were assessed based on baseline and post-intervention comparisons of dietary intake, physical activity, and screen time.

#### Anthropometric measurements

Fathers’ and adolescents’ body weight and height were measured separately twice by trained research staff in a private space using a digital scale (BWB-800 Scale, Tanita) and a stadiometer. Measurements were completed using standardized procedures of the National Health and Nutrition Examination Survey (NHANES) [51]. Fathers’ body mass index (BMI) was calculated using weight (kg) divided by height squared (m^2^). Adolescent BMI percentiles for age and sex were generated by a SAS program based on the 2000 CDC Growth Charts [52].

#### Father energy balance-related behaviors

##### Food intake

Fathers’ food intakes including fruit, vegetables, sugar-sweetened beverages (SSBs), sweets/salty snacks, and fast food, were measured using an adapted food behavior checklist [53]. The food behavior checklist showed internal consistency (*r* = 0.80, *p* < 0.05) in a study with low-income nutrition education program participants [53]. To assess fathers’ fruit/vegetable consumption, one question each was used separately about fruit and vegetable intake, “How many servings of fruits (vegetables) do you eat each day?” with blank lines for participants to write in numbers of servings. To examine fathers’ SSB consumption, two questions were used: “Do you drink fruit drinks, sports drinks, or punch?” and “Do you drink regular soda?”. To assess consumption of sweet/salty snacks, two questions were used: “Do you eat candy, ice cream, or other sweets or desserts?” and “Do you eat chips, puffs, or other salty snacks?”. Fast food intake was assessed by asking fathers one question: “Do you eat fast foods from fast-food restaurants such as Pizza Hut, McDonald’s, or Taco Bell?” Response options for questions regarding SSBs, sweets/salty snacks and fast food were no = 1, yes sometimes = 2, yes often = 3, and yes always = 4. Responses to the two questions for both SSBs and sweets/salty snack intakes were summed and averaged to create a score.

##### Physical activity

Fathers’ physical activity level was assessed using the Godin-Shephard Leisure-Time Physical Activity Questionnaire [54, 55]. An initial question was open-ended: “How many times on average do you do the following kinds of exercise for more than 15 minutes during your free time in a week?” with a blank line to write a number based on times per week for each category: strenuous exercise, moderate exercise, and mild exercise. A validation study showed adequate correlations between questionnaire results and percentile VO_2_ max and percentile body fat, and acceptable test-retest reliability [54].

##### Screen time

Fathers’ sedentary behaviors were assessed using two media use questions from the Project EAT survey [56]. The questions were “In your free time on an average weekday, how many hours do you spend doing the following activities?” and “In your free time on an average weekend day, how many hours do you spend doing the following activities?” Each question included four activities: “(1) watching TV/DVD/Videos, (2) using a computer (not for work), (3) playing electronic games while sitting, and (4) using smartphones or tablets” with response options for each activity: 0 hours, 0.5 hours, 1 hour, 2 hours, 3 hours, 4 hours, and 5+ hours. Total screen time hours per day were calculated by first summing weekday screen time hours plus weekend day screen time hours based on a sum of time spent on the four activities per day, followed by dividing the sum of weekly screen time hours by 7 days [56]. Screen time was top coded for fathers who reported > 10 hours of screen time per day (*n* = 2 and 3 at baseline and post data collection, respectively). This cut-off point was determined because of the distribution of participants’ responses and responses indicating unreasonable reporting and/or multi-tasking [57].

#### Adolescent energy balance-related behaviors

##### Dietary intake

In-person and phone 24-hour dietary recall interviews using Nutrition Data System for Research software (NDSR) (Nutrition Coordinating Center, University of Minnesota) were conducted to estimate adolescent dietary intake. NDSR 24-hour dietary recall interviews showed sufficient accuracy and validity in two studies with fourth- and third-grade children [58, 59]. The goal in the current study was for each adolescent to have one recall in person and two recall interviews over the phone within the next 1-2 weeks. The majority of adolescents had at least two dietary recall interviews (76% for baseline and 79% for post) with about half completing three recalls (53% for baseline and 54% for post). Recall interview days were selected to balance the distribution of weekdays and weekend days for participants.

For the recall interviews, adolescents were asked to report all foods, beverages, and water that they consumed in the last 24 hours. A Food Amounts Booklet, which showed illustrations of foods or abstract shapes and figures in different sizes, was provided to assist in estimating amounts consumed. Intakes were averaged across the number of recalls per adolescent and servings per day were determined for five food groups. Fruit and vegetable intakes were calculated separately using the NDSR fruit category total and vegetable category total (excluding fried vegetables and fried potatoes). Intake of SSBs was calculated based on the NDSR beverage category including sweetened soft drinks, fruit drinks, tea, coffee, coffee substitute, and water. Sweets/salty snack intake was calculated using foods from several NDSR categories, including chips and other salty snacks, meat and vegetable-based snacks, ready-to-eat cereals, grain-based desserts, dairy desserts, candies, sugars, jams, syrups, and sweet sauces. Intake of fast-food servings was calculated using foods from several NDSR categories, including fried chicken, fish, and shellfish (as a commercial entrée and fast food restaurants), and fried vegetables and fried potatoes from any source. Serving sizes for foods in the NDSR database were based on Dietary Guidelines for Americans recommendations when available or Food and Drug Administration serving sizes [60].

##### Physical activity and screen time

Adolescent physical activity level was assessed by a question, “In a usual week, how many hours do you spend doing the following activities” in three categories [(1) vigorous exercise, (2) moderate exercise, (3) mild exercise] [54, 56]. Moderate to vigorous activity questions showed adequate reliability (test-retest *r* = 0.73) among diverse adolescents in a Project EAT study [61]. Each category included response options “none, less than 30 minutes, 30 minutes-2 hours, 2.5-4 hours, 4.5-6 hours, and 6+ hours” with specific examples for activities in each category. To estimate adolescents’ physical activity hours per week, response options were coded as follows: none = 0, less than 30 minutes = 0.3, 30 minutes-2 hours = 1.3, 2.5-4 hours = 3.3, 4.5-6 hours = 5.3, and 6+ hours = 8. To create a score regarding total leisure time spent being physically active in a usual week, adolescents’ coded responses in the three categories were summed. Adolescents’ screen time was assessed in the same manner as fathers. Top coding for adolescents who reported > 10 hours of screen time per day was done for *n* = 18 and 9 at baseline and post data collection, respectively).

### Data analysis

All fathers with baseline data were included in the analysis of the fathers’ food and activity behavioral outcomes. Similarly, all adolescents with baseline data were included in analysis of adolescents’ food and activity behavioral outcomes. .

Baseline descriptive statistics for father and adolescent demographic and household characteristics, overall and by randomization group, include means and standard deviations for continuous variables, and count and percentages for categorical variables.

Baseline descriptive statistics of father EBRBs (dietary intake, physical activity, and screen time) and BMI outcomes and adolescent EBRBs and BMI percentile outcomes, overall and by randomization group, include means and standard deviations for these continuous outcomes.

#### Fathers’ energy balance-related behaviors and BMI outcome models

Linear regression mixed models were used to evaluate differences in mean change from baseline to post-intervention in father EBRB and BMI outcomes between the intervention and delayed-treatment control groups. The mixed models were adjusted for adolescent sex and age and included a random intercept for site and a random intercept for fathers nested within sites to account for clustering of fathers within sites. Per protocol sensitivity linear regression mixed models were used for father EBRBs and BMI outcomes; the per protocol models defined group assignment by participation group instead of by randomization group and were limited to fathers of adolescents with both baseline and post-intervention data.

#### Adolescents’ energy balance-related behaviors and BMI percentile outcome models

Linear regression mixed models were used to evaluate differences in mean change from baseline to post-intervention in adolescent EBRBs and BMI percentile between the intervention and delayed-treatment control groups. All models were adjusted for adolescent sex and age and included a random intercept for site and a random intercept for adolescent nested within site to account for clustering of adolescents within sites. Per protocol sensitivity linear regression mixed models for adolescent EBRB and BMI percentile outcomes were also used; the per protocol models defined group assignment by participation group instead of by randomization group and were limited to adolescents with both baseline and post-intervention data.

EBRB and BMI mixed model residual errors were evaluated for normality using the Shapiro-Wilk test for normality, which can be sensitive to small deviations from normality in large samples. Linear regression mixed models were used for evaluation of between group differences in mean outcome measure change even if the assumption of normally distributed model residuals was violated as estimates can be biased when outcomes are transformed, and violation of the residual normality assumption does not noticeably impact results when the number of observations per variable is > 10 [62].

Data analysis was conducted using SAS software version 9.4 (Cary, NC, USA, 2002–2012) with statistical significance defined as *P* < 0.05.

## Results

**Fig. 2:**
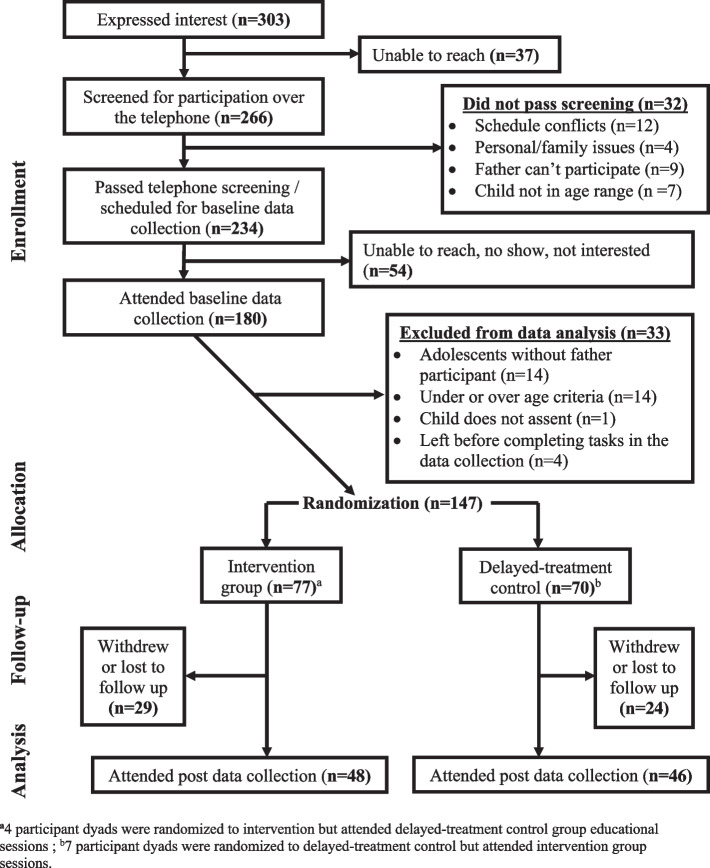
CONSORT diagram (Father/adolescent dyads)

A total of 303 father-adolescent dyads expressed interest in participating in the study (Fig. 2). Of those, 266 were screened for eligibility via a telephone interview, and 234 were identified as eligible. Data were not available from 54 father-adolescent dyads who did not attend the baseline data collection session and 33 dyads were excluded from the data analysis due to age criteria, absence of father, incomplete assent, and left before completing tasks in the baseline data collection. At the baseline data collection sessions, a total of a 147 father/adolescent dyads were randomized to intervention (*n* = 77) or delayed-treatment control (*n* = 70). Of those, 11 participants did not correctly follow the random assignment. Four participant dyads randomized to intervention attended the delayed-treatment control group educational sessions while 7 participant dyads randomized to delayed-treatment control group attended the intervention group. Thus, 80 participant dyads attended intervention group educational sessions and 67 participant dyads attended delayed-treatment control group educational sessions.

Of 147 randomized father/adolescent dyads, a total of 94 fathers and 102 adolescents completed post data collection resulting in a retention rate of 64% for fathers and 70% for adolescents. Eight youth attended post-data collection sessions with their mothers only. Those who did not complete post-intervention data collection withdrew from the study because of relocation or scheduling conflicts or were lost to follow up (unable to contact).

A sample size calculation showed that a sample of 96 father-youth dyads would be needed in each group (intervention and delayed-treatment control groups) after accounting for 20% attrition from the randomized *n* = 120 dyads in each group. This sample size was needed for observation of a 0.5 SD effect size for intake of SSBs (a decrease of 0.21 servings) with > 90% power to detect this change as significant [63, 64]. Restrictions on in-person interactions because of the COVID-19 pandemic in March 2020 resulted in an inability to continue to implement the program in person and, thus, a smaller sample size than anticipated.

The analysis sample includes 147 adolescent-father dyads with baseline data; 77 were randomized to the intervention group, 70 to the delayed treatment control group (Table 1).

**Table 1:** Adolescent and father demographic characteristics

Adolescent and father characteristics	All ***n*** = 147^**a**^	Intervention ***n*** = 77	Control ***n*** = 70
**Adolescent characteristics**			
Age, mean years (SD^b^)	12.2 (1.4)	12.2 (1.5)	12.2 (1.4)
Age distribution, n (%)			
10	37 (25.2)	21 (27.3)	16 (22.9)
11	34 (23.1)	18 (23.4)	16 (22.9)
12	31 (21.1)	14 (18.2)	17 (24.3)
13	25 (17.0)	13 (16.9)	12 (17.1)
14	20 (13.6)	11 (14.3)	9 (12.9)
Sex, n (%)			
Male	79 (54.1)	43 (55.8)	36 (52.2)
Female	67 (45.9)	34 (44.2)	33 (47.8)
BMI^b^ percentile^c^, mean (SD^b^)	78.5 (23.9)	81.5 (20.1)	75.1 (27.3)
**Father characteristics**			
Age, mean years (SD^b^)	41.7 (7.3)	42.5 (7.5)	40.7 (7.1)
Annual income, n (%)			
< $25,000	61 (43.6)	37 (50.0)	24 (36.4)
$25,000 - < $50,000	59 (42.1)	32 (43.2)	27 (40.9)
≥ $50,000	20 (14.3)	5 (6.8)	15 (22.7)
Marital status, n (%)			
Married	121 (84.6)	66 (86.8)	55 (82.1)
Living with partner	10 (7.0)	4 (5.3)	6 (9.0)
Single /widowed /divorced/separated	12 (8.4)	6 (7.9)	6 (9.0)
Education, n (%)			
Middle school or less	56 (38.9)	34 (44.2)	22 (32.8)
High school grad or GED^b^	59 (41.0)	26 (33.8)	33 (49.3)
College (any) or technical school	29 (20.1)	17 (22.1)	12 (17.9)
Employment, n (%)			
Self-employed	22 (15.6)	10 (13.3)	12 (18.2)
Unemployed	6 (4.3)	3 (4.0)	3 (4.6)
Part time employment	12 (8.5)	9 (12.0)	3 (4.6)
Full time employment	101 (71.6)	53 (70.7)	48 (72.7)
Years in US, n (%)			
< 10	4 (2.8)	3 (4.1)	1 (1.5)
10 - < 20	76 (53.9)	38 (51.4)	38 (56.7)
20 - < 30	52 (36.9)	27 (36.5)	25 (37.3)
≥ 30	9 (6.4)	6 (8.1)	3 (4.5)
Language, n (%)			
More Spanish than English	116 (80.6)	59 (77.6)	57 (83.8)
Equal Spanish and English	25 (17.4)	16 (21.1)	9 (13.2)
More English than Spanish	3 (2.1)	1 (1.3)	2 (2.9)
Father BMI^b^ (kg/m^2^), mean (SD^b^)	29.2 (3.7)	29.4 (4.1)	29.0 (3.3)

^a^N indicated for characteristics with missing data

^b^*SD* standard deviation, *GED* general education development, *BMI* body mass index

^c^Adolescent BMI percentiles for age and sex were calculated from SAS codes based on the 2000 CDC Growth Charts

The mean (SD) age of all adolescents was 12.2 (1.4) years. The majority of all adolescents were male (54%) with 46% female. Mean (SD) BMI percentile of all adolescents was 78.5 (23.9). Overall, the mean (SD) father age was 41.7 (7.3) years. Most fathers (86%) reported a yearly household income of < $50,000. About 39% of fathers completed middle school or less, 41% completed high school or GED, and 20% completed college or technical school. Approximately 72% of the fathers were employed full-time, and 85% were married. Most fathers reported speaking exclusively or primarily Spanish at home (81%) and having lived in the U.S. for more than 10 years (97%). The mean BMI of all fathers was 29.2 (3.7) kg/m^2^.

### Father EBRB and mean BMI outcomes

Baseline descriptive statistics for father EBRBs and mean BMI; overall and for intervention and delayed-treatment control groups are reported in Table S3.

No significant differences in mean baseline to post-intervention changes were observed for father EBRBs or BMI between intervention and delayed-treatment control groups in the adjusted mixed models (Table 2). Two non-significant results in the desired direction were observed between intervention and delayed-treatment control groups for father SSB (*p* = 0.0670) and fast food (*p* = 0.0903) intakes.

**Table 2:** Adjusted group differences for baseline to post change in FATHER EBRB outcomes and BMI

Baseline to Post change	Estimate (SE^**a**^) and ***p***-value for fixed effects from mixed model^**b**^ with random intercept for site and random intercept for father nested within site			
	Group^**c**^ (Ref = Control)	Time^**d**^ (Ref = Baseline)	Group*time^**e**^ (Ref = Control Baseline to Post change)	***P*** values for group*time
EBRBs^a^				
Fruit servings/day	0.31 (0.20)	0.43 (0.19)	− 0.28 (0.25)	0.282
Vegetable servings/day	0.06 (0.20)	0.24 (0.19)	0.18 (0.26)	0.458
SSB^a^ frequency^f^	0.09 (0.08)	−0.01 (0.07)	− 0.19 (0.10)	0.067
Sweets/salty snacks frequency^f^	0.01 (0.08)	− 0.03 (0.05)	−0.10 (0.08)	0.196
Fast food frequency^f^	− 0.04 (0.08)	−0.01 (0.07)	− 0.16 (0.09)	0.090
Physical activity times/week	0.10 (0.72)	0.21 (0.65)	1.37 (0.90)	0.134
Screentime^g^ hrs/day	0.63 (0.41)	0.63 (0.35)	−0.31 (0.49)	0.526
BMI^a^ kg/m^2^	0.43 (0.71)	0.57 (0.66)	0.68 (0.89)	0.445

^a^*Abbreviations*: *SE* standard error, *EBRB* energy balance related behaviors, *BMI* body mass index, *SSB* sugar sweetened beverage

^b^Models were adjusted for child age and sex

^c^Group effect estimates the adjusted difference between intervention and control means across both times

^d^Time effect estimates the adjusted difference between baseline and post means across both groups

^e^Group*time estimates the adjusted difference in mean change from baseline to post for intervention compared to control, none of the differences in adjusted baseline to post changes between intervention and control were significant at *p* < 0.05

^f^Frequency was a score based on 1-2 items per category and response options of never, sometimes, often, and always

^g^Screen time hours calculated for those with at least 6 of 8 screen time items

### Adolescent EBRB and BMI percentile outcomes

Baseline descriptive statistics for adolescent EBRBs and BMI percentile; overall and by intervention and delayed-treatment control groups are reported in Table S4. No significant differences in mean baseline to post-intervention changes were observed for adolescent EBRB or BMI percentile outcomes between intervention and delayed-treatment control groups in the adjusted mixed models (Table 3).

**Table 3:** Adjusted group differences for baseline to post change in ADOLESCENT EBRB outcomes and BMI percentile

Baseline to Post change	Estimate (SE^**a**^) and ***p***-value for fixed effects from mixed model^**b**^ with random intercept for site and random intercept for adolescent nested within site			
	Group^**c**^ (Ref = Control)	Time^**d**^ (Ref = Baseline)	Group*time^**e**^ (Ref = Control Baseline to Post change)	***P*** values for group*time
EBRBs^a^				
Fruit servings/day	− 0.04 (0.22)	0.09 (0.22)	− 0.02 (0.30)	0.947
Vegetable servings/day	−0.12 (0.22)	−0.14 (0.25)	0.44 (0.35)	0.209
SSB^a^ servings/day	−0.10 (0.11)	−0.11 (0.12)	0.10 (0.17)	0.556
Sweets/salty snacks servings/day	−0.08 (0.24)	−0.15 (0.26)	0.27 (0.36)	0.453
Fast food servings/day	0.16 (0.14)	0.04 (0.15)	0.15 (0.21)	0.475
Physical activity hrs/day	0.02 (0.09)	0.21 (0.08)	−0.10 (0.11)	0.363
Screen time^f^ hrs/day	0.95 (0.47)	0.02 (0.38)	−0.13 (0.53)	0.806
BMI^a^ percentile	6.69 (4.00)	−0.81 (0.60)	−0.87 (0.83)	0.295

^a^*Abbreviations*: *SE* standard error, *EBRB* energy balance related behaviors, *BMI* body mass index, *SSB* sugar sweetened beverage

^b^Models were adjusted for child age and sex

^c^Group effect estimates the adjusted difference between intervention and control means across both times

^d^Time effect estimates the adjusted difference between baseline and post means across both groups

^e^Group*time estimates the adjusted difference in mean change from baseline to post for intervention compared to control, none of the differences in adjusted baseline to post changes between intervention and control were significant at *p* < 0.05

^f^Screen time hours calculated for those with at least 6 of 8 screen time items

Mixed model residuals did not meet Shapiro-Wilk test criteria for normality for any father or adolescent EBRB or BMI outcomes except father fruit intake outcome; linear models were used to avoid estimate bias related to transformation of outcome variables [62]. Sensitivity analysis mixed model results limited to adolescents with both baseline and post-intervention data and their fathers, and with intervention and control groups defined by per protocol participation instead of by random assignment were similar (Analysis sample *n* = 94 dyads; 48 Intervention group, 46 delayed-treatment control group). There were no significant differences in mean change between intervention and delayed treatment control groups for any adolescent EBRB or BMI percentile outcomes nor for any father EBRB or BMI outcomes.

## Discussion

This study examined whether fathers’ and adolescents’ EBRBs and weight status were improved after they completed the Padres program (*n* = 147). No reductions were observed in mean consumption of SSBs and fast food by intervention group fathers compared to delayed-treatment control group fathers (*p* = 0.067 and *p* = 0.090, respectively). No significant differences in mean change in father and adolescent EBRBs and weight status were observed between the intervention and delayed-treatment control groups. Thus, the present study did not support the primary study hypothesis.

Calories consumed from beverages slightly increased among Hispanic adults in the US between 1977 and 2012, with SSBs ranked as the third highest source of snacks (kilocalories per capita per day), respectively, according to the findings from dietary recalls of eight nationally representative surveys [65]. SSBs were shown to have little nutritional value and linked to weight gain and dental caries as well as obesity and obesity-related health problems including metabolic syndrome, type 2 diabetes, coronary health disease, and stroke in the U.S. and worldwide [66–70]. While not significant at the *p* < 0.05 level, the current study showed results in the desired direction regarding lowering fathers’ SSBs intake from baseline to post-intervention, consistent with the evaluation of a family-based intervention (Familias Unidas for Health and Wellness (FUHW), where 12% of parents were fathers [71]. The FUHW intervention promoted healthy eating and activity strategies in overweight Hispanic adolescents and their parents and resulted in a reduction in added sugar intake among Hispanic parents [71]. This study addressed a gap in the literature by involving Latino fathers as the primary research participants in an intervention program to improve EBRBs for fathers and adolescents.

The Padres program showed no significant intervention effects on Latino adolescent EBRBs and BMI percentile and on father EBRBs and BMI. The lack of significant changes could be because the program was effective, but the study was underpowered or the program was ineffective. Other reasons that could explain the lack of significant changes may be limited resources that constrained access to healthy foods and physical activity opportunities needed to implement intervention strategies. Structural inequities in food access and affordability can impact healthy eating behaviors for people of color in the U.S. [72]. Based on the Hispanic Community Children’s Health/Study of Latino Youth (HCHS/SOL) between 2012 and 2014, 42% of Hispanic/Latino youth (ages 8-16) lived in food-insecure households, and 10% lived in a severe food-insecure household where family members experienced hunger [73]. In the current study, 39% of fathers reported that they were food insecure, and about 86% reported annual incomes less than $50,000. Thus, limited resources may have constrained the ability to make healthy food and activity behavior changes among Latino families in the current study.

Another possible explanation for lack of significant changes in adolescent EBRBs after participation in the Padres program may be the lack of time to practice improved behaviors prior to post-intervention data collection. Adolescent post-intervention measurements (diet, physical activity and weight status) were completed 1 week after the final session with additional 24-hour dietary recalls conducted 2-3 weeks after the final session. Because dietary intake can vary substantially from day to day, short-term success in making positive dietary changes regarding fat, fiber, fruit and vegetables, and sodium was defined as consistent change for several weeks or months, while long-term success was defined as change for 6 months to 1 or more years [74]. Thus, measuring dietary and activity behaviors soon after the final session may not adequately document potential short- or long-term success. Three months of follow-up data after completing the intervention in the current study were not included in the analysis because of the limited number of families participating in the 3-month data collection sessions. Changing living situations and other life events pose ongoing challenges to research in community-based settings.

There were several limitations of this study. The study findings may not be generalizable to the broader Latino population because participants of the Padres program were from one geographic location (Minneapolis/St. Paul metropolitan area), represented several Latin American countries, and most were from low-income households. Most of the data including EBRBs were self-reported by fathers and adolescents. Participants might over-report healthy behaviors and under-report unhealthy behaviors due to poor recall or social desirability. In-person program delivery was discontinued in March 2020 because of the COVID-19 pandemic, resulting in a smaller sample size than planned for according to the sample size calculations and limited power to detect significant changes. Randomization assignments were not followed correctly for eleven dyads which could have introduced bias into the group comparisons since groups were defined by randomization assignment, not by group participation. Because the intervention was community-based, some participants within sites may have known each other, and therefore, preferred to attend sessions based on attending with their friends, neighbors, and relatives at more convenient times or when they could share transportation. However, sensitivity analysis results comparing groups defined by participation instead of by randomization were similar. Because participants may have known each other, delayed-control participants may have been exposed to the intervention, which is a potential limitation. Finally, some of the fathers and adolescents who participated in the study might have enrolled because of their interest in nutrition and wellbeing, which would make them different from the general population.

## Conclusions

A community-based, culturally, and linguistically appropriate intervention focused exclusively on low-income, urban Latino fathers/male caregivers and their adolescents showed some results that were not statistically significant at the *p* < 0.05 level, but showed encouraging results in the desired direction for fathers. No intervention effects were observed for EBRBs among adolescents.

## Supplementary Information

**Additional file 1.**

**Additional file 2.**

**Additional file 3.**

**Additional file 4.**

**Additional file 5.**

## Data Availability

The de-identified data and SAS codes used in this study are available on request from the corresponding author. The data are not publicly available because while the intervention and all data collection has been completed, the data analysis phase of the study is still currently being completed.

## Abbreviations

EBRB: Energy balance-related behaviors
Padres program: Padres Preparados, Jóvenes Saludables
BMI: Body mass index
SSBs: Sugar-sweetened beverages
NDSR: Nutrition Data System for Research software
