# Supplementary material for: Padres Preparados, Jóvenes Saludables: intervention impact of a randomized controlled trial on Latino father and adolescent energy balance-related behaviors
Source: BMC Public Health. 2022 Oct 18;22:1932. doi: 10.1186/s12889-022-14284-5 (PMC9578196; doi:10.1186/s12889-022-14284-5)
Supplement: Supplementary file 1 — Additional file 1. [file 12889_2022_14284_MOESM1_ESM.docx]

**Table S1** Padres Preparados, Jóvenes Saludables of session structure, content, and participants

| Food preparation and family meal (all participants)  Side dish recipes included veggie sticks and dip, mango salsa, guacamole, fruit-infused water, veggie toppings, fruit kabobs |
| --- |
| Introduction to lesson activities (all participants) |
| Interactive segment part 1 (parents and youth separately)  Parents - hopes and dreams for adolescents, parenting styles and practices, parenting across multiple cultures, adolescent developmental concepts, communication skills, rules, conflict management, monitoring and supervision, parent-adolescent connection (parents)  Adolescents - hopes and dreams, how multiple cultures affect lifestyle choices, decision-making, communication skills, rules and choices, peer influence, family connection |
| Physical activity (all participants)  Indoor cardio, dance, chair yoga, relays, agility ladder |
| Interactive segment part 2 (all participants)  Parents and adolescents – energy balance concepts, recommendations and choices around fruit and vegetables, sugar sweetened beverages, sweets/salty snacks, fast food, physical activity and screen time, identifying and addressing barriers to healthy choices, label reading, marketing, meal and snack planning, purchasing healthy foods |
| Interactive segment part 3 (parents and youth separately)  Parents – apply parenting practices to adolescent EBRBs (setting expectations, role modeling, making healthy choices available) through role play, scenarios  Adolescents – activities to further interact with lesson content around healthy food and activity behaviors |
| Review and goal setting (all participants) |
