# Supplementary material for: Padres Preparados, Jóvenes Saludables: intervention impact of a randomized controlled trial on Latino father and adolescent energy balance-related behaviors
Source: BMC Public Health. 2022 Oct 18;22:1932. doi: 10.1186/s12889-022-14284-5 (PMC9578196; doi:10.1186/s12889-022-14284-5)
Supplement: Supplementary file 2 — Additional file 2. [file 12889_2022_14284_MOESM2_ESM.docx]

| **Table S2** Padres Preparados, Jóvenes Saludables session topics and objectives |
| --- |

| **Topics** | **Adolescent objectives** | **Parent objectives** |
| --- | --- | --- |
| **Session 1** Positive parenting and healthy habits | Articulate why maintaining a healthy lifestyle and healthy self-image is important.  Reflect on their current eating and physical activity behaviors.  Recognize the influence of eating and physical activity behaviors on energy balance.  Value youth and parent roles in healthy lifestyle as a family. | Assess family use of energy balance-related behaviors (EBRBs) to support health.  Describe importance of EBRBs for adolescent health goals.  Identify key elements of a positive parenting style that will help parents encourage EBRBs.  Recognize and reflect on how parenting styles influence adolescent EBRBs. |
| **Session 2** Multiple cultures and active lifestyles | Recognize the benefits of navigating multiple worlds and having a strong family connection.  Explain how physical activity contributes to energy balance and the importance of meeting recommendations.  Identify strategies with parent involvement to be physically active.  Set and monitor goals for meeting physical activity recommendations with parents. | Reflect upon immigrant experience and implications for adolescent health and physical activity.  Appreciate and identify attitudes and skills associated with navigating across different cultures and effects on EBRBs.  Explain the recommendations for and benefits of physical activity and contribution to energy balance.  Apply parenting practices to increase adolescent physical activity. |
| **Session 3** Adolescent development and healthy eating | Reflect on decision-making processes to make healthy eating and physical activity decisions.  Explain how fruits and vegetables contribute to energy balance and the importance of increasing fruit and vegetable intake.  Identify strategies with parent involvement to increase fruit and vegetable consumption.  Set and monitor goals to meet recommendations for fruit and vegetable consumption with parents. | Reflect on experience as adolescents and how adolescents are experiencing adolescence.  Explain teen brain development and function, how it differs from adults, and implications for parenting practices.  Explain the three stages of adolescent development, the purpose of adolescence and impact of family stress.  Identify the benefits of eating fruits / vegetables, recommended portion sizes, and daily intake.  Apply parenting practices to increase adolescent fruit and vegetable intake. |
| **Session 4** Communication and limiting screen time | Apply positive communication skills.  Explain how screen time influences energy balance and the importance of reducing screen time.  Apply strategies with parent involvement to manage screen time.  Set and monitor goals of screen time activities with parents. | Explain specific positive communication skills that promote mutual respect and trust between parents and adolescents.  Explain the benefits of reducing and limiting screen time for parents and adolescents.  Identify strategies to help teens reduce and limit screen time using parenting practices.  Role play the use of active listening skills and “I” messages when reducing and limiting screen time with adolescents. |
| **Session 5** Family rules and healthy beverages | Explain how sugary drinks influence energy balance and the importance of reducing intake of sugary drinks.  Select healthy beverages in different settings.  Identify and apply strategies with parent involvement to overcome barriers to reducing sugary drink consumption.  Set and monitor goals of limiting sugary drink consumption with parents. | Explain the importance of establishing clear negotiable and non-negotiable rules for adolescents.  Distinguish between punishment and discipline.  Describe how to use positive reinforcement as a tool for discipline and relationship building.  Explain the benefits of limiting sugar sweetened beverages for parents and adolescents and set goals to limit intake.  Use sugar sweetened beverage label information and parenting practices to influence adolescent and parent beverage selection. |
| **Session 6** Managing conflicts and healthy snacks | Recognize the importance of setting and following rules in families.  Explain how sweets and salty snacks influence energy balance and the importance of eating healthy alternatives.  Identify and apply strategies with parent involvement to select healthy snack options in different settings.  Set and monitor goals of limiting sugary snacks. | View conflict as a normal part of adolescent growth and parenting.  Develop collaborative conflict/anger management strategies.  Explain the health benefits of limiting sweets and salty snacks for parents and adolescents.  Use information on food labels to make healthy snack choices.  Apply key parenting practices to limit intake of sweets/salty snacks. |
| **Session 7** Supervision and fast food | Manage peer influence to support healthy behaviors.  Explain how fast food influences energy balance and the importance of reducing fast food intake.  Identify and apply strategies with parent involvement to overcome barriers to reducing fast food consumption.  Set goals and monitor the consumption of fast food with parents. | Define and explain the importance of monitoring and supervising teenager’s time and behavior.  Distinguish the levels of supervision based on age, environment and personality.  Identify health benefits of limiting fast food and portion sizes.  Apply key parenting practices and monitoring strategies to limit and reduce fast food intake.  Identify strategies for teens to handle peer pressure to eat at fast food restaurants and/or make unhealthy food choices. |
| **Session 8** Family connection and family meals | Explain importance of family meals and how family meals contribute to energy balance.  Appreciate importance of achieving energy balance via healthy lifestyle. behaviors with parent involvement.  Recognize youth’s role in leading healthy lifestyles in the family. | Describe the importance of parent-adolescent bonding and how to overcome barriers to strong bonds.  Practice skills for bonding with adolescents and responding to their bid for connection.  Explain health benefits of increasing family meal frequency.  Describe “healthy” family meals and plan menus for 3 family meals for next week.  Apply key parenting practices to increase frequency of family meals. |
