# Supplementary material for: Padres Preparados, Jóvenes Saludables: intervention impact of a randomized controlled trial on Latino father and adolescent energy balance-related behaviors
Source: BMC Public Health. 2022 Oct 18;22:1932. doi: 10.1186/s12889-022-14284-5 (PMC9578196; doi:10.1186/s12889-022-14284-5)
Supplement: Supplementary file 3 — Additional file 3. [file 12889_2022_14284_MOESM3_ESM.docx]

**Table S3** Father baseline outcome measures

| **Outcomes^1^** | **All**  **n = 147^2^** | **Intervention**  **n = 77** | **Control**  **n = 70** |
| --- | --- | --- | --- |
| **Diet Outcomes, mean (SD)** |  |  |  |
| Fruit intake, serving/day; n=128 | 2.18 (1.13) | 2.31 (1.15) | 2.02 (1.08) |
| Vegetable intake, serving/day; n=131 | 1.98 (1.22) | 2.01 (1.23) | 1.95 (1.22) |
| SSB^3^ intake, frequency; n=144 | 1.91 (0.46) | 1.95 (0.44) | 1.87 (0.49) |
| Sweets and salty snack^3^ intake, frequency; n=146 | 1.95 (0.51) | 1.96 (0.52) | 1.94 (0.49) |
| Fast food^3^ intake, frequency; n=146 | 2.00 (0.50) | 1.99 (0.50) | 2.01 (0.50) |
| **Weekly times per week of physical activity^4^ and daily screen time^5^ outcomes, mean (SD)** |  |  |  |
| Physical activity, times/week; n=126 | 5.60 (3.92) | 5.67 (4.09) | 5.52 (3.74)) |
| Screen time, hours/day; n=145 | 3.26 (2.41) | 3.57 (2.47) | 2.91 (2.30) |
| **BMI outcomes, mean (SD)** |  |  |  |
| Father BMI, kg/m^2^; n=142 | 29.21 (3.74) | 29.41 (4.10) | 29.00 (3.34) |

**^1^**Two-sample t-test of difference in means; ^2^N reported for each outcome; ^3^Response options (1=no, 2=yes, sometimes, 3= yes, usually, 4=yes, always); ^4^More than 15 minutes counts 1 time; ^5^Top coded at 10 hours / day, screen time hours for 3 was calculated from 6-7 items missing 1-2 of 8 item.
