# Supplementary material for: Padres Preparados, Jóvenes Saludables: intervention impact of a randomized controlled trial on Latino father and adolescent energy balance-related behaviors
Source: BMC Public Health. 2022 Oct 18;22:1932. doi: 10.1186/s12889-022-14284-5 (PMC9578196; doi:10.1186/s12889-022-14284-5)
Supplement: Supplementary file 4 — Additional file 4. [file 12889_2022_14284_MOESM4_ESM.docx]

**Table S4** Adolescent baseline outcome measures

| **Outcomes^1^** | **All**  **n = 147^2^** | **Intervention**  **n = 77** | **Control**  **n = 70** |
| --- | --- | --- | --- |
| **Number of NDSR^3^ diet recalls, mean (SD);** n = 144 | 2.3 (0.8) | 2.3 (0.9) | 2.4 (0.8) |
| **Diet outcomes, mean (SD)** |  |  |  |
| Fruit intake, NDSR serving/day; n = 143 | 1.53 (1.25) | 1.52 (1.15] | 1.54 (1.37) |
| Vegetable intake, NDSR serving/day; n = 144 | 1.62 (1.25) | 1.54 (1.37) | 1.70 (1.12) |
| SSB intake, NDSR serving/day; n = 143 | 0.47 (0.64) | 0.42 (0.54) | 0.52 (0.72) |
| Sweets/salty snack intake, NDSR serving/day; n = 143 | 1.64 (1.41) | 1.61 (1.32) | 1.68 (1.51) |
| Fast food intake, NDSR serving/day; n = 143 | 0.40 (0.76) | 0.47 (0.84) | 0.31 (0.65) |
| **Daily hours of physical activity^4^ and screentime^5^ outcomes, mean (SD)** |  |  |  |
| Physical activity, hours/day; n = 147 | 0.62 (0.57) | 0.62 (0.57) | 0.61 (0.58) |
| Screen time, hours/day; n = 146 | 4.97 (3.03) | 5.38 (3.00) | 4.51 (3.05) |
| **BMI outcome, mean (SD)** |  |  |  |
| BMI percentile; n = 138 | 78.5 (23.9) | 81.5 (20.1) | 75.1 (27.3) |

**^1^**Two-sample t-test of difference in means; ^2^ N reported for each outcome; ^3^NDSR= Nutrition Data System for Research; ^4^Top coded at 2 hours / day ^5^Top coded at 10 hours / day, screen time hours for 3 missing 1-2 of 8 items was calculated from 6-7 items.
