# Supplementary material for: Padres Preparados, Jóvenes Saludables: intervention impact of a randomized controlled trial on Latino father and adolescent energy balance-related behaviors
Source: BMC Public Health. 2022 Oct 18;22:1932. doi: 10.1186/s12889-022-14284-5 (PMC9578196; doi:10.1186/s12889-022-14284-5)
Supplement: Supplementary file 5 — Additional file 5. [file 12889_2022_14284_MOESM5_ESM.docx]

# **Figure S1**

# **Padres Preparados Jovenes Saludables Program Trial Protocol**

## **Project summary**

Objective: To improve Latino youth (10-14 years) energy balance-related behaviors (EBRBs) (fruit, vegetable, sugar-sweetened beverage, sweets/salty snack and fast-food intakes, physical activity, screen time) by increased frequency of positive paternal parenting practices.

Design: A randomized controlled study with a delayed-treatment control group and randomization at the father-youth dyad. Outcome data will include dietary intake, physical activity/screen time, anthropometric measures, and parenting practice frequency collected at baseline, immediately post, and 3-months post-intervention.

Setting: Community organizations and churches in Minneapolis/St. Paul, MN.

Participants: 40 dyads at each of 6 sites between September 2017-February 2021.

Intervention: An existing 8-week parenting curriculum will be adapted using community-based participatory research principles to promote positive parenting practices (modeling, availability, expectations).

Main Outcome Measures: Youth EBRBs measured with 24-hr dietary recalls/questionnaires, BMI-for-age percentile from measured height/weight. Independent variables include group, time, and group x time interaction.

Analysis: Multiple regression models to test the null hypothesis that changes in outcomes from baseline to immediately post and 3-months post intervention will be the same in intervention and control groups on an intention-to-treat basis. Baseline outcomes, covariates to adjust for baseline differences, potential confounders, and site effect will be included in the models. Statistical significance will be set at p < 0.05, two-tailed.

## **General information**

**Protocol title**

Padres Preparados Jovenes Saludables: The *Latino Fathers Promoting Healthy Youth Behaviors*

**Sponsor/funder**

This project is supported by the National Institute of Food and Agriculture, U.S. Department of Agriculture, under award number 2016-68001-24921.

**Figures and tables**

All the tables and figures provided in Figure S1: Padres Preparados Jovenes Saludables Program Trial Protocol are supplemental tables and figures.

## **Rationale & background information**

A family resiliency framework for Latino families has been described based on the integration of cultural values, beliefs, and practices within the context of how individual, family and community resilience intersect.^1^ The centrality of family (familism) in the life of Latinos, family involvement and support are salient family resilience factors contributing to parental relationships that serve as strong protective factors for Latino youth. Therefore, incorporating the concept of familism into obesity prevention interventions by involving fathers and youth in a shared program may contribute to better health behaviors.^2^

The prevalence of obesity and overweight among Hispanic youth in the U.S. is higher than other population groups contributing in disparities in disease risk. National Health and Nutrition Examination Survey (NHANES) data (2013-16) showed that the prevalence of obesity for youth 2-19 years was 23.6% for Hispanic, 14.7% for non-Hispanic White, and 9.8% for Non-Hispanic Asian youth.^3^ A review by Ruiz et al.^4^ (2020) indicated that adverse cardio-metabolic outcomes were more prevalent among obese compared to normal weight youth including hypertension, dyslipidemia, and insulin resistance and type 2 diabetes (Ruiz et al. 2020). Among Hispanic adolescents (12-18 years), NHANES data (2005-2016) indicated a prevalence of 22.5% for prediabetes, 16.5% for impaired fasting glucose, and 6.8% for increased glycohemoglobin.^5^

Energy balance-related behaviors (EBRBs) among youth include greater intake of foods with low energy density such as fruits and vegetables and intake of energy-dense foods such as processed snack foods, sweets and sugar sweetened beverages (SSBs).^6^ According to NHANES (2009-2014) data, the total mean HEI-2015 score for Mexican-American and other Hispanic adolescents (12-18 years) was about half of the maximum 100 total points (53.5 and 54.2, respectively),^7^ representing a need to improve diet quality based on the Dietary Guidelines for Americans (DGA) recommendations.^6^ Consumption of healthful foods by U.S. Hispanic adolescents (12-19 years) was well below 2020-2025 DGA daily recommendations^6^ based on NHANES 2017-2018 data^8^ for total fruit (0.9 of 1.5 recommended cup equivalents (eq), total vegetables (0.9 of 2-2.5 cup eq), whole grains (0.6 of 3 oz eq) and dairy (1.7 of 3 cup eq). Data from the 2011-2016 NHANES showed that on a given day, Mexican American and Other Hispanic adolescents (12-19 years) consumed 522 and 565 g (18 and 20 oz) of sugar-sweetened beverages, respectively.^9^ Data from the 2011-2012 NHANES showed that about 15% of calories on a given day were from fast food (restaurant fast food/pizza) among Hispanic youth (2-19 years).^10^ NHANES data 2005-2016 showed that Mexican American/Hispanic adolescents had a larger snack size (kcal/snacking occasion) and higher mean daily intake of added sugars compared to white adolescents.^11^

Reviews have shown inverse associations between physical activity and adolescent obesity, while screen time was positively associated with overweight/obesity in children < 18 years.^12-14^ Physical activity recommendations for school-aged youth (6-11 years) include 60 minutes of daily moderate-to-vigorous physical activity and no more than 2 hours/ day of screen-time viewing.^15^ Based on a sample of US Hispanic/Latino youth aged 8 to 16 years (n = 1466) in the Study of Latino Youth, time spent in moderate-vigorous activity was below recommendations with 25 minutes of moderate and 10 minutes of vigorous activity and 605 minutes of sedentary activity per day as measured by interviews and accelerometers.^16^

Application of behavioral theory to dietary/physical activity interventions can provide information about specific variables that influence frequency of desirable behaviors and thus affect intervention effectiveness.^17, 18^ Social Cognitive Theory (SCT) has commonly been used as a framework for interventions to modify dietary and physical activity behaviors of youth based on the identification of personal and environmental variables predictive of behaviors.^17^ Important variables include parenting practices that alter the physical and social environment enabling positive behavior changes among youth.^19^ Food parenting practices that influence EBRBs among youth include modeling, setting expectations and managing availability of foods and beverages.^20^ Food parenting practices, socioeconomic status and food security have been associated with BMI and obesogenic dietary intakes among Latino children and adolescents.^21, 22^

Mothers have traditionally had primary responsibility for providing food to children as their primary caregiver. Therefore, much of the evidence for relationships between food parenting practices and youth diet behaviors is based on information from mothers reported in studies involving younger children.^20^ However, two recent reviews have addressed fathers’ influence on diet behaviors of youth^23, 24^ indicating a growing interest based on an expanding role for fathers as caregivers. A systematic review of fathers’ influence on youth diet behaviors (birth to 18 years) included five studies indicating that diet quality of fathers and children’s overall intake were positively correlated.^23^ Several studies also found that fathers’ use of restriction, control over food choices, and pressure to eat had mostly negative impacts on child eating behavior. Results from a narrative review of fathers’ involvement in child feeding indicated that fathers influence children’s intake in a positive (through modeling) and negative manner (use of coercive feeding practices, which differs from mothers) during mealtimes.^24^

The body of literature related specifically to Latino fathers and parenting practices regarding EBRBs for older children is limited. A review by O’Connor et al.^25^ included five qualitative studies which indicated that in Latino households, mothers had primary responsibility for food decision-making around youth intake as it related to food provisioning and preparation. Several cross-sectional studies supported the concept that Latino fathers are involved in their children’s dietary intake through various food parenting practices. Use of controlling and reinforcing practices by Latino fathers of children (7-13 years) was associated with lower and greater fruit and vegetable intake among children, respectively.^26^ Another study showed that Mexican American fathers of children 8-10 years were more likely to use practices based on control and pressure than mothers, while mothers’ and fathers’ practices scores were correlated to a modest/moderate extent. ^27^ Limited studies have addressed ways to improve these practices through father-focused interventions.

Studies have shown that Latino fathers are involved in food parenting practices that influence intake of older children and adolescents,^25-27^ yet intervention studies with Latino families have primarily focused on mothers.^28^ Ash et al.^28^ reviewed the literature regarding family-based obesity prevention interventions that addressed diet, physical activity or both published from 2008 to 2015 and after 2015. Of those conducted in the U.S., 17 studies primarily involved Hispanic/Latino families. Seven studies included parent-child dyads or parents and children where the majority of parents involved were mothers (range 71% – 98%) and children were in the preschool to adolescent age range. In another 5 studies only mothers and children from preschool to 13 years were involved. Several other studies did not identify the distribution of parents or primary caregivers by mother or father involvement or the study only described methodology.

A community based participatory research (CBPR) approach can empower communities to be involved in decision-making processes and influence program design, implementation and evaluation.^29^ Thus, the likelihood that interventions will reach participants in a culturally respectful and appropriate manner is increased. CBPR recognizes that building on community strengths and relationships is critical to successful research implementation.

Intervention programs are needed that address obesity prevention and healthy behaviors for a growing immigrant population in the United States,^30^ and target fathers of early adolescents, a group generally not actively involved in family intervention programs, yet is widely recognized for playing a key role in healthy youth development.

## **Study goals and objectives**

Therefore, the purpose of this project is to adapt, implement and evaluate efficacy of a curriculum specifically for Latino fathers and youth (10-14 years), using CBPR principles that incorporate parenting skills education to increase frequency of positive paternal parenting practices (role modeling, availability, expectations, communication) to improve youth EBRBs and weight status (10-14 years). A randomized, controlled trial (RCT) will be conducted to test the hypotheses that compared to a delayed-treatment control group immediate post-intervention and 3 months post-intervention, statistically significant changes will be observed in a) the frequency of positive paternal food and physical activity parenting practices, and b) EBRBs including greater fruit and vegetable intake, lower intake of SSBs, sweets/salty snacks, and fast food, and greater physical activity and less screen time, and stable weight status among youth.

## **Study design**

During the first year of the project, an existing Latino parenting skills curriculum^31^ will be adapted, pilot tested and revised as needed based on input from a stakeholder advisory team, a father advisory board, and a series of focus group interviews with fathers, mothers and youth. During the following 4 years (between September 2017-February 2021), an RCT will be conducted at several sites in conjunction with community partners in a staggered fashion based on implementation of the adapted curriculum, followed by data analyses and reporting.

Latino fathers will be recruited through Latino-serving organizations and churches in several urban communities and one rural community. Inclusion criteria are being a father or other male caregiver who is a 1^st^ generation immigrant, able to speak Spanish, present at mealtime with the adolescent in the study for at least one meal per day three days per week, and at least one youth in the home (10-14 years) who will participate with the father in the intervention program. Father/adolescent dyads are the primary research participants, mothers will also be welcome to attend program sessions.

## **Methodology**

The *Latino Fathers Promoting Healthy Youth Behaviors* (Padres Preparados Jovenes Saludables) program will be adapted from an existing *Padres Informados (Padres*) curriculum developed to prevent tobacco and other substance use among Latino youth (age 10-14) through a family-skills training program.^31^ The previous curriculum was implemented with primarily Latino mothers and youth. *Padres* resulted in lower smoking susceptibility among youth in families with less adherence to traditional Latino cultural values^2^ and was effective across most parenting outcomes at 6 months post intervention.^32^

The obesity prevention program for Latino fathers and youth will be built on Social Cognitive Theory^33^ (SCT) (Figure 1) which supports the need to address youth EBRBs with parenting practices that improve the home food and physical activity environment, provide positive observational learning opportunities, and focus on effective parent communication skills. Figure 1 was adapted to present the reciprocal determinism construct from a family perspective.^34^ Potential learning goals/objectives are summarized in Table 1 based on the existing *Padres* curriculum which focused on parenting skills/styles including communication, conflict management, developing parent-youth connections, monitoring and parenting across cultures. Learning objectives and instructional activities will be developed using CBPR methods,^29^ and will utilize the existing curriculum (*Padres*)^31^, and other curricula, materials and resources available. A Father Advisory Board will be convened to review and comment on adaptations of the curriculum and implementation of the project to focus on engaging fathers.

**ENVIRONMENT**

Physical/social environmental variables

- **Parenting practices**

- Modeling

- Home availability

- Rules/expectations

- Communication

- General parenting style

Primary: Dietary and physical activity behaviors of youth

Secondary: Frequency of paternal parenting practices

Demographic characteristics

Cultural considerations

**BEHAVIOR (parent/child)**

- Eating practices/ patterns
- Home food practices/

food management

- Physical activity practices

**INDIVIDUAL (parent/child)**

- Self-efficacy
- Outcome expectations
- Preferences
- Behavioral capability

Child BMI

**Fig S1**. A social cognitive/reciprocal determinism framework of influences on child food and physical activity behavior

**Tab S1**. Potential Session Objectives and Activities for Parents and Joint Parent/Child Activities

| Title | Objectives for Parent Sessions | Activities for parenting portion (parents) | Activities for EBRB portion (parents and children together) | Theory-based Determinants Addressed |
| --- | --- | --- | --- | --- |
| Session 1: Parenting practices and healthy habits | Assess family use of Energy Balance-Related Behaviors (EBRBs) to support health.  Describe importance of EBRBs for children’s health and future goals.  Identify key elements of a positive parenting style that will help parents encourage EBRBs.  Recognize and reflect on how their own parenting styles influence their children’s EBRBs. | Assessment of parenting style by parent  Introduction to 3 primary parenting practices (modeling, availability, expectations) | Assessment of current EBRBs and comparison to recommendations | Problem identification  Goal setting  Outcome expectations  Behavioral capability |
| Session 2: Multiple cultures, living an active lifestyle | Reflect upon their immigrant experience and implications for their children’s health and physical activity.  Appreciate and identify attitudes and skills associated with navigating across different cultures and effects on EBRBs.  Explain the recommendations for and benefits of physical activity and contribution to energy balance.  Apply family-based and parenting practices to increase child physical activity. | Acculturation grid to identify level of acculturation for parent and child  Application of parenting practices to improve child physical activity | Benefits and barriers to physical group activity (identify and share barriers and ways to overcome them in family groups) | Problem identification, Self-efficacy  Outcome expectations  Preferences  Behavioral capability  Goal setting |
| Session 3: Adolescent development and healthy foods | Reflect on their experience as adolescents and how their children are experiencing adolescence.  Explain teen brain development and function, how it differs from adults, and implications for parenting practices for EBRBs.  Explain the three stages of adolescent development, the purpose of adolescence and impact of family stress.  Identify the benefits of eating fruits/vegetables, recommended portion sizes, and daily intake.  Apply three parenting practices to increase children’s fruit and vegetable intake. | Puzzle activity to explore parent reactions to child development  Application of parenting practices to improve child fruit and vegetable intake | MyPlate assessment of fruit and vegetable intake, identification of barriers to intake and ways to overcome them | Outcome expectations  Self-efficacy  Preferences  Behavioral capability  Goal setting |
| Session 4: Communication and limiting screen time | Explain specific positive communication skills that promote mutual respect and trust between parents and youth.  Explain the benefits of reducing and limiting screen time for parents and children.  Identify strategies to help teens reduce and limit screen time using parenting practices.  Role play the use of active listening skills and “I” messages when reducing and limiting screen time with their children. | Active listening and I-messages activities  Application of parenting practices to limit child screen time (role plays) | Assessing screen time  Developing a family media plan to limit screen time | Outcome expectations  Self-efficacy  Behavioral capability  Goal setting |
| Session 5: Rules, expectations and healthy beverages | Explain the importance for adolescents of establishing clear negotiable and non-negotiable rules.  Distinguish between punishment and discipline.  Describe how to use positive reinforcement as a tool for discipline and relationship building.  Explain the benefits of limiting sugar sweetened beverages for self and child and set goals to do so.  Interpret labels on SSBs and use this information and parenting practices to influence child and parent beverage selection. | Parenting pyramid, 5-1 rule  Application of parenting practices to limiting child sugary drink intake | Activities to determine calories and grams sugar in various beverages, relationship to EB, cost (interactive stations) | Outcome expectations  Self-efficacy  Behavioral capability  Goal setting |
| Session 6: Managing conflicts and healthy snacks | View conflict as a normal part of adolescent growth and parenting.  Develop collaborative conflict and anger management strategies.  Explain the health benefits of limiting sweets and salty snacks for self and child.  Use information on food labels to make healthy snack choices.  Apply key parenting practices to limit sweets/salty snacks. | Problem solving in a collaborative way to manage conflicts  Application of parenting practices to limit child intake of sweets and salty snacks | Portion size activities, identification of healthy snack alternatives | Outcome expectations  Self-efficacy  Behavioral capability  Goal setting |
| Session 7: Monitoring / supervision and fast food | Define and explain the importance of monitoring and supervising teenager’s time and behavior.  Distinguish the levels of supervision based on age, environment and personality.  Identify the health benefits of limiting fast food and portion size for self and their teenagers.  Apply key parenting practices and monitoring strategies to limit and reduce fast food.  Identify strategies for teens to handle peer pressure to eat at fast food restaurants and/or make unhealthy food choices. | Communication strategies/ parents as coaches (Supervision Soccer Game)  Application of parenting practices to limit child fast food intake | Demonstration of ways to limit fast food intake, and calories from fast food | Outcome expectations  Self-efficacy  Behavioral capability  Goal setting |
| Session 8: Connecting with your child and family meals | Describe the importance of parent-child bonding and how to overcome barriers to strong parent-child bonds.  Practice skills for bonding with one’s teenager and responding to their bid for connection.  Explain health benefits of increasing frequency of family meals.  Describe “healthy” family meals and plan 3 for next week.  Apply key parenting practices to support increasing frequency of family meals. | Rocks and beans activity, language of encouragement activity, Group discussion to identify barriers to family meals, ways to overcome barriers.  Application of parenting practices to increase family meal frequency. | Relay activity to identify benefits of family meals | Outcome expectations  Self-efficacy  Behavioral capability  Goal setting |

A project team will conduct 4 focus groups (urban and at least one rural site) with a convenience sample of fathers from the community to discuss the adaptation of the content and the delivery of the program. A focus group protocol will guide a systematic method of inquiry to promote a dialectic discussion. The project team will work closely with the community agency collaborators throughout the project. The multiple channels of ongoing communication with collaborators will inform the adaptation of the *Padres* project curriculum as will best practices in adapting prevention/intervention programs for Latino audiences.^35^ The curriculum will include: 1) a trainer’s manual of eight class sessions, 2) a manual of eight food preparations sessions, 3) instruction on eight 15-minute joint physical activity breaks, and 4) a set of goal setting homework to encourage father and youth implementation of skills.

**Pilot testing**

Based on the feedback received in the focus groups and the Father Advisory Board, the curriculum will be pretested in a pilot study. Eight sessions will be implemented at one community organization. Each session will be two and a half hours in duration, with a total of 24 contact hours with each father and youth. The research team will develop and finalize research protocols; prepare data collection instruments and methods; train local implementation staff in research and evaluation methods, human subjects’ protections, and data security; and work with the Stakeholder Advisory Team to ensure the cultural compatibility of the intervention, research design and data collection methods.

**Intervention implementation**

The program will be implemented at collaborating sites in a staggered fashion with recruitment by community sites/agencies that are known and trusted within the Latino community including community-based organizations, extension sites, public schools and churches to enhance recruitment and retention.^36^ Collaborating sites will be reimbursed for their staff time to participate in the project including a parent-trainer who will lead the implementation of the intervention and a site recruitment coordinator. Staff who lead the curriculum sessions are expected to be bilingual Latino fathers themselves and will undergo training regarding parenting practices involved in youth EBRBs and instructional strategies about teaching and learning including group management. Participants will be compensated with cash or gift cards to maximize recruitment and retention.

Twenty (20) parent/youth dyads will attend each of six series of eight weekly sessions. Outcome data will be collected at baseline (prior to session 1), immediate post-intervention (after session 8), and 3 months post-intervention to determine changes in youth EBRBs and weight status, and paternal parenting practices.

The study statistician will use SAS to generate separate randomization schedules for each site. The randomization schedules will be created with randomized blocks of 2, 4, and 6. Within each block, assignments will be balanced between Intervention and Control to ensure approximately equal group size if the complete randomization schedule isn't used.  Random assignments will be printed on colored paper slips, folded 3 times and placed in sequentially numbered opaque envelopes to be distributed to enrolled families by the project coordinator. Eventually, father and youth dyads will be randomized (following block randomization procedures) to intervention or control conditions following completion of outcome data assessments at baseline.

**Measurement**

Progress toward outcomes will be measured utilizing standard, validated instruments (Table 2). Some are available in Spanish and others will be translated and back-translated by the project team. A description of the outcome and measurement instruments follows.

**Tab S2.** Summary of assessment measures

| **Assessment** | **Assessment method and participants** |
| --- | --- |
| Demographic characteristics and anthropometric measurements | Questionnaire on sociodemographic characteristics (father)  Self-reported birthdate and sex (adolescent)  Height, weight measurements^59^ (father/adolescent) |
| Dietary behavioral outcomes | 24-hour dietary recall interviews using Nutrition Data System for Research software^54, 55^ (adolescent)  Food Behavior Checklist^57, 58^ (father)  Skin carotenoid scores assessed with reflection spectroscopy (Veggie meter)^56^ (father)  Frequency of family meals^64^ (father/adolescent) |
| Physical activity and screen time-related behavioral outcomes | Physical activity frequency questions from several sources^61, 62^ (adolescent)  Physical activity questions (time estimate) (Godin-Shepherd Leisure-Time Exercise Questionnaire)^61^ (father)  Screen time questions (time estimate)^62^ (father/adolescent) |
| Parenting practices outcomes | Father parenting practices measures^28^ for fruit, vegetable, sugar sweetened beverage, sweets/salty snack and fast food intake, physical activity and screen time (father/adolescent) |
| Father specific influences on adolescent dietary intake | Father meal/food involvement (planning, buying, preparing with child - created for trial) (father/adolescent)  Father self-efficacy questions for promoting healthy eating and physical activity^65^ (father)  Parenting styles questions^67-69^ (father/adolescent)  Communication^66^ (father) |
| Home food environment | Home food availability questions from Project EAT^63^(father/adolescent) |

Youth will participate in 3 24-hour dietary recall interviews^37^ using Nutrition Data System for Research (NDSR) software version 2016^38^ to assess change in intake of low energy dense foods (fruits and vegetables) and high energy dense foods (sugar-sweetened beverages, sweets/salty snacks, and fast foods). Recalls will be collected in-person during the in-person data collection sessions, followed by 2 recalls by phone within the next week. Recalls will be collected on 3 non-consecutive days including 2 weekdays and 1 weekend day. A food amounts booklet will be provided to help adolescents identify quantities consumed. Skin carotenoid levels will also be assessed using reflection spectroscopy with a Veggie Meter as a biomarker of vegetable and fruit intake.^39^ Fathers will complete the Food Behavior Checklist developed and tested for low-income, low literacy adults^40, 41^ to assess intake frequency of fruits, vegetables, sugar sweetened beverages, sweets/salty snacks, and fast food.

Height and weight will be assessed according to established anthropometric methods^42^ using a stadiometer (model: Seca 202, Hanover, MD) and a digital weight scale (model: Tanita BWB-800P Digital Medical Scale, Arlington Heights, IL, USA) to assess whether BMI changes over time among the intervention vs. control group. Three measures of both height (to the nearest 0.1 cm) and weight (to the nearest 0.1 kg) will be collected and averaged. BMI percentiles and z-scores for adolescents will be generated using a SAS program created by the Centers for Disease Control and Prevention.^43^

Adolescent physical activity will be assessed by a question “In a usual week, how many hours do you spend doing the following activities” in three categories [(1) vigorous exercise, (2) moderate exercise, (3) mild exercise].^45, 46^ Each category will include response options to quantify time with specific examples for activities in each category. Adolescent sedentary behaviors will be assessed using two media use questions from the Project EAT survey:^46^ “In your free time on an average weekday, how many hours do you spend doing the following activities?” and “In your free time on an average weekend day, how many hours do you spend doing the following activities?” for 4 activities: (1) watching TV/DVD/Videos, (2) using a computer (not for homework), (3) playing electronic games while sitting, and (4) using smartphones or tablets. Response options for each activity will quantify sedentary time. Father physical activity level will be assessed using the Godin-Shephard Leisure-Time Physical Activity Questionnaire^45, 47^ based on the question “How many times on average do you do the following kinds of exercise for more than 15 minutes during your free time in a week?” with a blank line to write a number based on times per week for each category: strenuous exercise, moderate exercise, and mild exercise. A validation study showed adequate correlations between questionnaire results and percentile VO_2_ max and percentile body fat and acceptable test-retest reliability.^45^ Father screen time will be assessed in the same manner as adolescents.^46^

Fathers and youth will complete questionnaires to assess frequency of paternal modeling, availability and expectations regarding fruit, vegetable, sugar-sweetened beverage, sweets/salty snacks and fast-food intakes based on existing questions tested for criterion validity.^28^ Assessment of the home food environment will be made to measure change in availability of healthy and less healthful foods^48^ as an indication of paternal food parenting practices. Family meal frequency which provides opportunity for father meal involvement will be measured using a single frequency item regarding frequency of eating meals together with family members.^49^ Change in confidence (self-efficacy) for promoting healthy eating and physical activity among adolescents will be assessed among fathers (completely disagree = 1 to completely agree = 5) using questions analogous to those used in the previous Aventuras Para Ni$ñ$os parent survey based on acceptable internal consistency.^50^ Father-youth communication will be assessed using a14-item parent-youth communication scale validated with a low-income largely African American population will be adapted and translated to Spanish.^51^ Parenting style will be assessed with survey items of general parenting primarily adapted from the Parenting Style Observation Rating Scale for Latino parents with additional items from the Parenting Style and Dimension Questionnaire and the Comprehensive General Parenting Questionnaire.^52-54^

At baseline only, a brief demographic questionnaire will be completed by fathers and adolescents to obtain information regarding age, sex, Hispanic/Latino heritage, years in the U.S., country of birth, household composition, education, employment, and participation in food assistance programs.

## **Safety considerations**

Procedures for recording and reporting adverse events and their follow-up will be developed. The project coordinator will collect information and manage reports.

## **Follow-up**

Outcome data will be collected at baseline (prior to session 1), immediate post-intervention (after session 8), and 3 months post-intervention to determine changes in youth EBRBs and weight status, and paternal parenting practices.

## **Data management**

First, data will be collected using paper-copy surveys, Qualtrics surveys, Veggie meter, and NDSR software in each study site. After each data collection, all of the data components will be entered by undergraduate students and cleaned and compiled by a graduate student.

1. **Description of Master Data File**

Baseline, post, and follow-up data of adolescents, fathers, and mothers and codebooks for adolescents' datasheets and fathers’ and mothers’ datasheets will be stored separately into sub sheets in the master data file. Adolescent baseline, post-, and follow-up datasheets will include survey data, intake data (collected by using NDSR), height/weight and birthdate data, and beta carotenoid scores data (collected by veggie meter), group assignments and randomization, and session attendance. Fathers’ and mothers' baseline, post-, and follow-up datasheets will include survey data, height/weight and birthdate data, and beta carotenoid scores data (collected by veggie meter), group assignments and randomization, and session attendance.

1. **Paper copy survey data**

Undergraduate and graduate students will enter the paper copy survey data twice into the two identical sheets in the excel file. A graduate student will compare these two identical sheets in the SAS and identify the data entry errors. For the unmatched variables, the graduate student will check the paper copy of the data and correct these variables on the sheet. These corrections will be logged into a tab named “Changes in compiled data” in the master data file. After all of the cleaning is done, the graduate student will compile the data into the data sheets of the master data file in the G-drive.

1. **Qualtrics survey**

Qualtrics data will be downloaded as a text format from Qualtrics. Based on the codebook of the datasheet, all of the options (text form) will be changed to the numeric form by using the find & replace function of excel. Finally, cleaned data will be compiled into the data sheets of the Master data file.

1. **NDSR food recall cleaning and compiling**

For the recalls, adolescents will be asked to report all foods, beverages, and water that they consumed in the last 24 hours. A Food Amounts Booklet will be provided to assist in estimating amounts consumed. All of the recalls will be reviewed by an NDSR certified person (either a graduate student or project coordinator) for data entry error. If there are any data entry errors, these errors will be corrected by the NDSR certified person. After this step, NDSR output files will be produced, and these files will be used in the SAS software to obtain intake data. In the SAS software, intakes will be averaged across the number of recalls, and unreliable recalls will be identified and deleted from the output data files. When all of the cleaning processes is done, NDSR recalls will be used to produce five food groups: fruit intake (servings per day), vegetable intake (servings per day), sugary drink intake (serving per day), sweets/salty snacks intake (serving per day), and fast-food intake (serving per day). The food components that comprised each NDSR food group are shown in Table 3. These five groups' intake data will be compiled into the data sheets of the master data file. Children’s HEI scores will be also calculated by using SAS software with NDSR output files.

**Tab S3.** NDSR food groups and their components.

| **Food groups** | **Food categories** |
| --- | --- |
| Fruit intake | Citrus juice  Fruit juice excluding citrus juice  Citrus fruit  Fruit excluding citrus fruit  Avocado and similar fruit  Fruit-based savory snacks |
| Vegetable intake | Dark-green vegetables  Deep yellow vegetables  Tomato  White potatoes  Other starchy vegetables  Legumes (cooked dry beans)  Other vegetables  Vegetable juice |
| Sugary drink intake | Sweetened soft drinks  Sweetened fruit drinks  Sweetened tea  Sweetened coffee  Sweetened coffee substitute  Sweetened water |
| Sweets/salty snacks | Meat-based savory snack  Frozen dairy desert  Frozen non-dairy desert  Pudding and other dairy desserts  Sugar  Syrup, honey, jam, jelly, preserves  Sauces, sweet-regular  Sauces sweet-reduced fat/reduced calorie/fat-free  Chocolate candy  Non-chocolate candy  Frosting or glaze  Vegetable-based savory snack  Crackers- whole grain  Crackers- some whole grain  Crackers- refine grains  Ready-to-eat cereal (presweetened)- whole grain  Ready-to-eat cereal (presweetened)- some whole grain  Ready-to-eat cereal (presweetened)- refined grain  Cakes, cookies, pies, pastries, Danish, doughnuts, cobblers – whole grain  Cakes, cookies, pies, pastries, Danish, doughnuts, cobblers – some whole grain  Cakes, cookies, pies, pastries, Danish, doughnuts, cobblers – refined grain  Snack bars- whole grain  Snack bars- some whole grain  Snack bars- refined grain  Snack chips- whole grain  Snack chips- some whole grain  Snack chips- refined grain  Popcorn  Flavored popcorn |
| Fast food | Fried vegetables  Fried potatoes  Fried chicken – commercial entrée and fast food  Fried fish - commercial entrée and fast food  Fried shellfish- commercial entrée and fast food |

## **Statistical analysis**

During the planning year, focus group interviews will be audio recorded and transcribed verbatim. A codebook will be developed, and transcripts will be coded by two independent project team members. A software program (NVivo v.12, QSR International (Americas) Inc., Burlington, MA) will be used to organize and enable analysis of coded transcripts. Facilitators and barriers to youth EBRBs and parenting practices will be identified using content analysis procedures based on recurring themes.^55^

Father and youth dyads assigned to the intervention condition (n = 120 dyads) will participate in the program immediately, while father and youth dyads assigned to the delayed-control condition (n = 120 dyads) will participate in the prevention program after the 3 months post intervention data collection session. This will allow detection of an effect size of 0.5 standard deviations in sugared-sweetened beverages (SSBs) 24-hr dietary recall with 90% power accounting for a 20% attrition rate (at 3 months post intervention follow-up).

Demographic characteristics of intervention and control group participants will be compared at baseline using t-tests for continuous variables and chi-square tests for categorical variables to assess whether participant characteristics were equivalent between groups. Adjustments for differences between groups will be done as needed. The primary outcomes, youth EBRBs, BMI-for-age percentile, and BMIz scores, will be evaluated by a multiple regression model (SAS software, SAS Institute Inc., Cary, NC) to test the null hypothesis that immediate post-intervention and 3-month post intervention outcomes are the same in the intervention and control groups. Baseline outcomes, covariates to adjust for baseline differences, potential confounders and site effect will be included in the regression model, to generate least squares adjusted mean changes, to generate a model comparing the intervention to control group. The secondary outcomes, self-reported parenting practices, will be evaluated by ANCOVA models to test the null hypotheses for each of the identified parenting behavior scale scores. Covariates to adjust for baseline differences, potential confounders and site effect will be included in the models to generate least squares adjusted mean changes for each group. A two-tailed significance level (alpha) of 0.05 will indicate statistically significant differences. The response rate expectation at 3 months post-intervention is 80% (based on 20% loss-to-follow up). This expected loss-to-follow up was accounted for in the power analysis for the primary outcome, which is based on 120 families per group. Multi-imputation of missing data is planned for the primary study outcome EBRBs. Chi-square tests and two-sample t-tests will be used to compare baseline characteristics between study completers and those lost to follow up to assess the potential for intervention effect bias due to study dropout. Secondary parenting outcome analyses will only include subjects with parenting practices scales data at the indicated data collection times for each analysis.

## **Quality assurance**

Weekly meetings among project staff and primary investigators will be held to assure quality. In these meetings, staff will talk about recruitment, program sessions, facilitators, data collection, and data management and report issues that affect recruitment, implementation, and data collection and management to maintain quality. Community partners will also be asked their feedback on sessions and data collections to assure quality.

## **Expected outcomes of the study**

**Primary outcome:** The primary outcomes include improvements in youth EBRBs and weight status from baseline to immediate post-intervention and 3 months post-intervention in the intervention compared to control group. Improvements in youth EBRBs include decreased intake of SSBs and sedentary activity and increased intake of fruits, vegetables, and breakfast consumption. The number of youths in the normal weight category according to BMI-for-age weight status categories (5th percentile to less than the 85th percentile) is expected to remain stable over the course of the intervention and the next 3 months.

**Secondary outcomes:** The secondary outcomes include increases in parenting behavior scale scores (parenting practices) between intervention and control parents immediate post-intervention and at 3 months post-intervention. These include making healthy foods and opportunities for physical activity available, role modeling positive EBRBs, setting expectations for improvements in youth EBRBs and improved father-child communication. To observe improvements in these secondary outcomes, fathers are expected to be more involved with their child’s eating and physical activity habits and mealtimes, and to use local resources to support improvements in youth EBRBs.

The project is designed to purposefully engage community organizations and participants based on CBPR^29^ principles in determining their own needs and learning preferences so that materials will be effective and lead to behavior change. The project brings together faculty and staff with relevant research and extension experience working with Latino families.

Immigrant communities, due to past trauma in their lives, often have a distrust of government and researchers or research practices.^56^ To address this potential issue, the project team will partner with community agencies that are respected in the community. Research materials and language will be carefully crafted with input from the Father Advisory Board to provide information in a respectful manner. The program will be implemented in conjunction with community collaborators using recruitment methods, which have been successful in the past. Therefore, difficulty recruiting the necessary number of father-early adolescent dyads is not expected. However, a higher attrition rate (20%) may occur. The project team will oversample to account for a higher attrition rate, compensate for time, and work with community agencies to implement the program at convenient times as suggested in a previous study involving programming for Latino immigrant families.^57^ The project team will record reasons for dropouts and address issues to enhance retention.

Accuracy of dietary recall by youth is subject to recall bias based on self-reported intake. However, collection of this information over three days (one day in person) using the NDSR multi-pass approach with visual portion-size estimation aids will minimize this error. In addition, parent input will be sought to answer questions about food products.

Participants will be enrolled from organizations in a single Midwestern state, therefore results may not generalize to Latino families in other regions of the US. However, research on Latino father populations in areas of recent settlement are rare, therefore this information may contribute new knowledge regarding how to prevent overweight or obesity in these rapidly growing but under-researched populations. Sampling bias may also occur because participants may have an interest in the subject matter or a higher level of motivation; therefore, results cannot be generalized to a broader group of parents.

Lastly, contamination may occur given that families being served by community organizations may know each other. Because the time investment in the sessions is significant, and sessions build on each other, parents are unlikely to casually share knowledge with other parents or convey substantive knowledge or skills. This issue can be addressed by discussing the problem with all service providers and asking participants to avoid sharing materials.

## **Dissemination of results and publication policy**

Results will be disseminated through the most effective method for the target audiences, including the use of print, video, Spanish language radio/TV broadcasts, web-based and social media technologies in English and/or Spanish. A detailed dissemination plan will be developed with input from the project team and collaborating agencies. This will ensure that information is returned via an effective communication method to the varied stakeholders who have an interest in results (i.e. study participants, collaborating organizations, community professionals, researchers, Extension, educators). Dissemination meetings will be held to present study main outcomes when the study is done.

Results and materials will be disseminated via Extension Just in Time Parenting CoP to which contributions will include webinars, information for the “front page”, and project reports and materials. The project team will also make presentations at scientific meetings and submit articles for publication in appropriate scientific journals.

Publication policy will be developed, and project team members will discuss about the topic and publication ideas and authorship and who will be acknowledge in the weekly meetings.

## **Duration of the project**

See figure 2 for the detailed timeline for the project for each site. In summary, after the baseline data collection tasks are completed by all family members who were participating, father and adolescent pairs will be given an envelope containing a random group assignment into either the treatment group or the waitlisted (delayed treatment) control group. The intervention group will be asked to attend the series of educational sessions the week after baseline data collection. The delayed-treatment control group will be asked to attend the series of educational sessions after the three-month data collection session.

**Fig S2.** Diagram of intervention procedures and timeline.

Weeks

| Treatment group | |  | 1 | 2 | 3 | 4 | 5 | 6 | 7 | 8 |  |  |  |  |  |  |  |  |  |  |  |  |  |  |  |  |  |  |  |  |  |  |
| --- | --- | --- | --- | --- | --- | --- | --- | --- | --- | --- | --- | --- | --- | --- | --- | --- | --- | --- | --- | --- | --- | --- | --- | --- | --- | --- | --- | --- | --- | --- | --- | --- |
|  | | D1 | Education sessions | | | | | | | | D2 | 12-week waiting | | | | | | | | | | | | D3 |  | | | | | | | |
| Delayed-treatment control group | |  |  |  |  |  |  |  |  |  |  |  |  |  |  |  |  |  |  |  |  |  |  |  | 1 | 2 | 3 | 4 | 5 | 6 | 7 | 8 |
|  | D1 | 8-week waiting | | | | | | | | D2 | 12-week waiting | | | | | | | | | | | | D3 | Education sessions | | | | | | | |  |

D1 = baseline data collection session

D2 = post data collection session

D3 = follow-up data collection session

## **Project timeline**

| Key: | * task implemented | | | | | | |  | T – training R - recruitment | | | | | | | | | | | |
| --- | --- | --- | --- | --- | --- | --- | --- | --- | --- | --- | --- | --- | --- | --- | --- | --- | --- | --- | --- | --- |
|  | ! deliverable | | | | | | |  | D - data collection | | | | | | | | | | | |
| Task/Milestones/  Deliverables |  | | | | | | | | | | | | | | | | | | | |
|  | **YEAR 1** | | | | **YEAR 2** | | | | **YEAR 3** | | | | **YEAR 4** | | | | **YEAR 5** | | | |
| QUARTER | 1 | 2 | 3 | 4 | 1 | 2 | 3 | 4 | 1 | 2 | 3 | 4 | 1 | 2 | 3 | 4 | 1 | 2 | 3 | 4 |
| *Project Development* |  |  |  |  |  |  |  |  |  |  |  |  |  |  |  |  |  |  |  |  |
| Recruit/ convene stakeholder team | * | * | * | * |  | * |  | * |  | * |  | * |  | * |  | * |  | * |  | * |
| Hire/assign staff | * |  |  |  |  |  |  |  |  |  |  |  |  |  |  |  |  |  |  |  |
| Finalize project site agreements | * |  |  |  |  |  |  |  |  |  |  |  |  |  |  |  |  |  |  |  |
| Convene collaborators/site representatives | * | * | * |  | * |  | * |  | * |  | * |  | * |  | * |  | * | * | * |  |
| Provide training to collaborators |  | * | * |  |  |  |  |  |  |  |  |  |  |  |  |  |  |  |  |  |
| Develop research protocols | * |  |  |  |  |  |  |  |  |  |  |  |  |  |  |  |  |  |  |  |
| IRB application/ approval | * | * |  |  |  |  |  |  |  |  |  |  |  |  |  |  |  |  |  |  |
| *Formative Research* |  |  |  |  |  |  |  |  |  |  |  |  |  |  |  |  |  |  |  |  |
| Convene parent advisory board | * | * |  |  |  |  |  |  |  |  |  |  |  |  |  |  |  |  |  |  |
| Develop, pilot & finalize curriculum |  | * | * | * |  |  |  |  |  |  |  |  |  |  |  |  |  |  |  |  |
| *Community Implementation* |  |  |  |  |  |  |  |  |  |  |  |  |  |  |  |  |  |  |  |  |
| Sites 1 & 2 |  |  | T | R | D | D | D |  |  |  |  |  |  |  |  |  |  |  |  |  |
| Sites 3 & 4 |  |  |  |  |  |  | T | R | D | D | D |  |  |  |  |  |  |  |  |  |
| Sites 5 & 6 |  |  |  |  |  |  |  |  |  |  | T | R | D | D | D |  |  |  |  |  |
| *Data analysis and reporting* |  |  |  |  |  |  |  |  |  |  |  |  |  |  |  |  |  |  |  |  |
| Data analysis |  |  |  |  |  |  |  |  |  |  | * | * | * | * | * | * | * | * |  |  |
| Final reports written and disseminated |  |  |  |  |  |  |  |  |  |  |  |  |  |  |  |  | * | * | * | ! |
| Report findings to stakeholders, collaborators, participants |  |  |  |  |  |  |  |  |  |  |  |  |  |  |  |  |  |  |  | * |
| Prepare & submit reports for AFRI |  |  |  |  | ! |  |  |  | ! |  |  |  | ! |  |  |  | ! |  |  |  |

## **Problems anticipated**

Immigrant communities, due to past trauma in their lives, often have a distrust of government and researchers or research practices. To address this potential issue, the project team will partner with community agencies that are respected in the community. Research materials and language will be carefully crafted with input from the Parent Advisory Board to provide information in a respectful manner.

The project is designed to purposefully engage stakeholders in determining their own needs and learning preferences so that materials will be effective and lead to behavior change. The project brings together faculty and staff with relevant research and extension experience working with Latino families. This capable and knowledgeable team will support a strong and effective effort that leads to the desired outcomes.

The program will be implemented in conjunction with community collaborators using recruitment methods which have been successful in the past. Therefore, difficulty recruiting the necessary number of father-early adolescent (pairs) is not expected. However, a higher attrition rate (20%) may occur. The project team will oversample to account for a higher attrition rate, compensate for time, and work with community agencies to implement the program at convenient times. The project team will record reasons for dropouts and address to enhance retention.

Potential hazards present in this project are related to personal interactions in community and travel. Activities of the project require travel to community settings. The U of MN works to minimize travel hazards by requiring every person who utilizes a U of MN vehicle to complete training. In addition, it is U of MN policy that all employees are required to carry appropriate insurance on their vehicles. Additional hazards may occur when at a community site. All project staff will review personal safety procedures before commencing community activities.

Monitoring for participant safety will be performed by the Principle Investigator and the U of MN Institutional Review Board (IRB). Monitoring by the IRB includes regularly providing information about enrollment, adverse events, protocol changes, and new literature in the field.

The nature of the work proposed is classified as low risk. In the case an adverse event would occur, site staff will be instructed to make appropriate referrals for care. Following these steps; study staff will call the Principle Investigator as soon as possible and provide detailed information on the incident. The Principal Investigator will make necessary referrals, and as appropriate, determine whether to stop the research. The Principle Investigator will also complete an adverse event report and submit to the IRB within three working days.

## **Ethics**

This study will be conducted according to the Declaration of Helsinki guidelines and approved by the University of Minnesota Institutional Review Board Human Subjects Protection Committee (reference: 1511S80707 and date of approval: 3 July 2016). The research study will be approved by the University of Minnesota Institutional Review Board with informed consent and assent for fathers and youth, respectively, and registered as a clinical trial. All parents will provide written consent and all adolescents will provide written assent to participate the study prior to the baseline data collection.

## **References**

1. Bermudez JM, Mancini JA. Familias Fuertes: Family Resilience among Latinos. In *Handbook of Family Resilience*. Becvar DS, Ed. New York, NY: Springer Science+Business Media, 2013.

2. Allen ML, Hurtado GA, Garcia-Huidobro D, Davey C, Forster J, Reynoso U, Alvarez de Davila S, Linares R, Gonzales N, Svetaz MV. Cultural contributors to smoking susceptibility outcomes among Latino youth: The Padres Informados/Jovenes Preparados participatory trial. *Fam Commun Health*. 2017;40:170-179. doi:[10.1097/FCH.0000000000000147](https://dx.doi.org/10.1097%2FFCH.0000000000000147)

3. Ogden CL, Fryar CK, Hales CM, Carroll MD, Aoki Y, Freedman DS. Differences in obesity prevalence by demographics and urbanization in US children and adolescents, 2013-2016. *JAMA*. 2018;319:2410-2418. doi:[10.1001/jama.2018.5158](https://dx.doi.org/10.1001%2Fjama.2018.5158)

4. Ruiz LD, Zuelch ML, Dimitratos SM, Scherr RE. Adolescent obesity: diet quality, psychosocial health, and cardiometabolic risk factors. *Nutrients*. 2020;12:43. doi: [10.3390/nu12010043](https://dx.doi.org/10.3390%2Fnu12010043)

5. Andes LJ, Cheng YJ, Rolka DB, Gregg EW, Imperatore G. Prevalence of prediabetes among adolescents and young adults in the United States, 2005-2016. *JAMA Pediatr*. 2020;174:e194498. doi:10.1001/jamapediatrics.2019.4498.

6. U.S. Department of Agriculture and U.S. Department of Health and Human Services. Dietary Guidelines for Americans, 2020-2025. 9th Ed. December 2020. <http://DietaryGuidelines.gov>. Accessed July 6, 2021.

7. Thomson JL, Tussing-Humphreys LM, Goodman MH, Landry AS. Diet quality in a nationally representative sample of American children by sociodemographic characteristics. *Am J Clin Nutr*. 2018;109-1-11.

8. USDA, ARS. *What We Eat in America*, NHANES 2015-2016, individuals 2 years and over (excluding breast-fed children), day 1 dietary intake data, weighted. Food Patterns Equivalents Database (FPED) 2017-2018. [www.ars.usda.gov/nea/bhnrc/fsrg](http://www.ars.usda.gov/nea/bhnrc/fsrg). Accessed July 6, 2021.

9. Russo RG, Northridge ME, Wu B. Characterizing sugar-sweetened beverage consumption for US children and adolescents by race/ethnicity. *J Racial and Ethnic Health Disparities.* 2020;7:1100–1116. https://doi.org/10.1007/s40615-020-00733-7

10. Vidraman S, Fryar CD, Ogden CL. Caloric intake from fast food among children and adolescents in the United States, 2011-2012. NCHS Data Brief. No. 213, September 2015. .<https://www.cdc.gov/nchs/data/databriefs/db213.pdf>. Accessed July 6, 2021.

11. Tripicchio GL, Kachurak A, Davey A, Bailey RL, Dabritz LJ, Fisher JO. Associations between snacking and weight status among adolescents 12–19 years in the United States. *Nutrients*. 2019;11:1486. doi:10.3390/nu11071486.

12. Carson V, Staiano AE, Katzmarzyk PT. Physical activity, screen time, and sitting among U.S. adolescents. *Pediatr Exerc Sci.* 2015;27:151-159. <https://doi.org/10.1123/pes.2014-0022>

13. Rauner A, Mess F, Woll A. The relationship between physical activity, physical fitness and overweight in adolescents: a systematic review of studies published in or after 2000. *BMC Pediatr*. 2013;13:19. https://doi.org/10.1186/1471-2431-13-19

14. Fang K, Mu M, Liu K, He Y. Screen time and childhood overweight/obesity: a systematic review and meta-analysis. *Child Care, Health Dev.* 2019;45:744-753. https//doi.org/10.1111/cch.12701

15. US Department of Health and Human Services. Physical Activity Guidelines for Americans. 2nd edition Washington, DC: US Department of Health and Human Services; 2018. <https://health.gov/sites/default/files/2019-09/Physical_Activity_Guidelines_2nd_edition.pdf>.

16. Evenson KR, Arredondo EM, Carnethon MR, Delamater AM, Gallo LC, Isasi CR, Perreira KM, Fote SA, Van Horn L, Vidot DC, Sotres-Alvarez D. Physical activity and sedentary behavior among US Hispanic/Latino youth: the SOL Youth Study. *Med Sci Sports Exerc.* 2019;51:891-899. Doi:10.1249/MSS0000000000001871.

17. Contento IR. *Nutrition Education: Linking Research, Theory, and Practice*. 2^nd^ ed. Sudbury, MA: Jones and Bartlett; 2011.

18. Diep CS, Chen TA, Davies VF, Baranowski JC, Baranowski T. Influence of behavioral theory on fruit and vegetable intervention effectiveness among children: a meta-analysis. *J Nutr Educ Behav*. 2014;46:506-46.

19. Vaughn AE, Ward DS, Fisher JO, Faith MS, Hughes SO, Kremers SPJ, Musher-Eizenman DR, O’Connor TM, Patrick H, Power TG. Fundamental constructs in food parenting practices: a content map to guide future research. *Nutr Rev*. 2016;74:98-117. doi: 10.1093/nutrit/nuv061

20. Yee AZH, Lwin MO, Ho SS. The influence of parental practices on child promotive and preventive food consumption behaviors: a systematic review and meta-analysis. *Int J Behav Nutr Phys Act.* 2017;14:47. doi: 10.1186/s12966-017-0501-3

21. LeCroy MN, Siega-Riz AM, Albrecht SS, Ward DS, Cai J, Perreira KM, Isasi CR, Mossavar-Rahmani Y, Gallo LC, Castañeda SF, Stevens J. Association of food parenting practice patterns with obesogenic dietary intake in Hispanic/Latino youth: results from the Hispanic Community Children’s Health Study/Study of Latino Youth (SOL Youth). *Appetite.* 2019;140:277-287. doi: [10.1016/j.appet.2019.05.006](https://dx.doi.org/10.1016%2Fj.appet.2019.05.006)

22. Ochoa A, Berge JM. Home environmental influences on childhood obesity in the Latino population: a decade review of literature. *J Immigr Minor Health.* 2017;19:430-447. Doi:10.1007/s10903-016-0539-3.

23. Litchford A, Savoie Roskos MR, Wengreen H. Influence of fathers on the feeding practices and behaviors of children: A systematic review. *Appetite*. 2020;147:104558. <https://doi.org/10.1016/j.appet.2019.104558>

24. Rahill S, Kennedy A, Kearney J. A review of the influence of fathers on children's eating behaviours and dietary intake. *Appetite*. 2020;147:104540. <https://doi.org/10.1016/j.appet.2019.104540>

25. O’Connor T, Perez O, Colón Garcia I, Gallagher M. Engaging Latino fathers in children’s eating and other obesity-related behaviors: a review. *Curr Nutr Rep*. 2018;7:29-38.

26. Parada H, Ayala GX, Horton LA, Ibarra L, Arredondo EM. Latino fathers’ feeding-related parenting strategies on children’s eating. *Ecol Food Nutr*. 2016;55:292-307.

27. Tschann JM, Gregorich SE, Penilla C, Pasch LA, de Groat CL, Flores E, Deardorff, Greenspan LC, Butte NF. Parental feeding practices in Mexican American families: initial test of an expanded measure. *Int J Behav Nutr Phys Act.* 2013;10:6. http://www.ijbnpa.org/content/10/1/6.

28. Ash T, Agaronov A, Young T, Aftosmes-Tobio A, Davison KK. Family-based childhood obesity prevention interventions: a systematic review and quantitative content analysis. *Int J Behav Nutr Phys Act*. 2017;14:113. <https://doi.org/10.1186/s12966-017-0571-2>

29. Arroyo-Johnson C, Allen ML, Colditz GA, Hurtado GA, Davey CS, Sanders Thompson VL, Drake BF, Svetaz MV, Rosas-Lee M, Goodman MS. A tale of two community networks program centers: operationalizing and assessing CBPR principles and evaluating partnership outcomes. *Prog Community Health Partnersh*. 2015;9 Suppl:61-9. DOI: 10.1353/cpr.2015.0026.

30. Pew Research Center. Factors on U.S. immigrants, 2018. Statistical portrait of the foreign-born population in the United States. <https://www.pewresearch.org/hispanic/2020/08/20/facts-on-u-s-immigrants/>.

31. Allen ML, Garcia-Huidobro D, Hurtado GA, Allen R, Davey C, Forster J, Hurtado M, Lopez-Petrovich K, Marczak M, Reynoso U, Trebs L, Svetaz MV. Immigrant family skills-building to prevent tobacco use in Latino youth: study protocol for a community based participatory randomized controlled trial. *Trials*. 2012;13:242.

32. Allen ML, Hurtado GA, Garcia-Huidobro D, et al. Cultural Contributors to Smoking Susceptibility Outcomes Among Latino Youth: The Padres Informados/Jovenes Preparados Participatory Trial. *Fam Community Health*. 2017;40(2):170-179. doi:10.1097/FCH.0000000000000147

33. Bandura A. Self-efficacy. In Ramachaudran VS, ed. *Encyclopedia of Human Behavior* (Vol. 4, pp. 71-81). New York, NY: Academic Press; 1994.

34. Cullen KW, Baranowski T, Rittenberry L, Cosart C, Hebert D, de Moor C. Child-reported family and peer influences on fruit, juice and vegetable consumption: reliability and validity of measures. *Health Educ Res.* 2001;16:187-200.

35. Bernal G, Bonilla J, Bellido C. Ecological validity and cultural sensitivity for outcome research: issues for the cultural adaptation and development of psychosocial treatments with Hispanics. *J Abnormal Child Psychol*. 1995;23:67-81.

36. Buscemi J, Blumstein L, Kong A, Stolley MR, Schiffer L, Odoms-Young A, Bittner C, Fitzgibbon ML. Retaining traditionally hard to reach participants: Lessons learned from three childhood obesity studies. *Contemp Clin Trials*. 2015;42:98–104.

37. Baxter SD, Hardin JW, Guinn CH, Royer JA, Mackelprang AJ, Smith AF. Fourth-grade children’s dietary recall accuracy is influenced by retention interval (target period and interview time). *J Am Diet Assoc*. 2009;109:846–856. <http://doi.org/10.1016/j.jada.2009.02.015>

38. University of Minnesota, Nutrition Coordinating Center, Nutrient Data System for Research Software. Available at: <http://www.ncc.umn.edu/products/>.

39. Ermakov IV, Gellermann W. Optical detection methods for carotenoids in human skin. *Arch Biochem Biophys*. 2015;572:101-111.

40. Townsend MS, Kaiser LL, Allen LH, Joy AB, Murphy SP. Selecting items for a food behavior checklist for a limited-resource audience. *J Nutr Educ Behav*. 2003;35:69–77.

41. Blackburn ML, Townsend MS, Kaiser LL, Martin AC, West EA., Turner B, Joy AB. Food behavior checklist effectively evaluates nutrition education. *California Agric*. 2008;60:20–24.

42. Lohman TG, Roche AF, Martorell R. *Anthropometric Standardization Reference Manual*; Champaign, IL: Human Kinetics Books; 1991.

43. Centers for Disease Control and Prevention, Division of Nutrition, Physical Activity, and Obesity. Growth Chart Training. A SAS Program for the 2000 CDC Growth Charts (ages 0 to <20 years) 2019.

[https://www.cdc.gov/nccdphp/dnpao/growthcharts/resources/sas.htm.](https://www.cdc.gov/nccdphp/dnpao/growthcharts/resources/sas.htm. )

45. Godin G, Shephard RJ. A simple method to assess exercise behavior in the community. *Can J Appl Sport Sci.* 1985;10:141–146.

46. Utter J, Neumark-Sztainer D, Jeffery R, Story M. Couch potatoes or french fries: Are sedentary behaviors associated with body mass index, physical activity, and dietary behaviors among adolescents? *J Am Diet Assoc.* 2003;103:1298-1305.

47. Godin, G. The Godin-Shephard leisure-time physical activity questionnaire. *Health Fitness J Canada.* 2011;4:18–22.

48. Widome R, Neumark-Sztainer D, Hannan PJ, Haines J, Story M. Eating when there is not enough to eat: eating behaviors and perceptions of food among food-insecure youths. *Am J Public Health*. 2009;99:822–828. doi: [10.2105/AJPH.2008.139758](https://dx.doi.org/10.2105%2FAJPH.2008.139758)

49. Fulkerson JA, Neumark-Sztainer D, Hannan PJ, Story M. Family meal frequency and weight status among adolescents: cross-sectional and 5-year longitudinal associations. *Obesity (Silver Spring).* 2008;16:2529-34.

50. Crespo NC, Elder JP, Ayala GX, Campbell NR, Arredondo EM, Slymen DJ, Baquero B, Sallis JF, McKenzie TL. Results of a multi-level intervention to prevent and control childhood obesity among Latino children: the Aventuras Para Ninos Study. *Ann Behav Med.* 2012;43:84–100. 10.1007/s12160-011-9332-7

51. Barnes H, Olson DH. Parent Adolescent Communication. In: Olson DH, McCubbin HI, Barnes H, Larson A, Muxen M, Wilson M, eds. *Family Inventories*. St. Paul, MN: Family Social Sciences; 1982:33-48.

52. Domenech Rodríguez MM, Donovick MR, Crowley SL. Parenting styles in a cultural context: observations of “protective parenting” in first-generation Latinos. *Fam Process.* 2009;48:195–210.

53. Olivari MG, Tagliabue S, Confalonieri E. Parenting style and dimensions questionnaire: a review of reliability and validity. *Marriage Fam Rev.* 2013:49:465–490.

54. Sleddens EFC, O’Connor TM, Watson KB, Hughes SO, Power TG, Thijs C, De Vries NK, Kremers SPJ. Development of the Comprehensive General Parenting Questionnaire for caregivers of 5-13 year olds. *Int J Behav Nutr Phys Act.* 2014;11:15.

55. Saldaña J. *The Coding Manual for Qualitative Researchers.* Sage; 2012.

56. Chavez-Dueñas NY, Adames HY, Perez-Chavez JG, Salas SP. Healing ethno-racial trauma in Latinx immigrant communities: Cultivating hope, resistance, and action. Am Psychol. 2019;74:*4*9-62. http://dx.doi.org/10.1037/amp0000289

57. Garcia-Huidobro D, Allen M, Rosas-Lee M, Maldonado F, Gutierrez L, Svetaz MV, Wieling E. [Understanding attendance in a community-based parenting intervention for immigrant Latino families.](https://www.ncbi.nlm.nih.gov/pubmed/25869496) *Health Promot Pract*. 2016;17:57-69. doi: 10.1177/1524839915582155.
